# *BmBlimp-1* gene encoding a C2H2 zinc finger protein is required for wing development in the silkworm *Bombyx mori*

**DOI:** 10.7150/ijbs.34743

**Published:** 2019-10-12

**Authors:** Song-Yuan Wu, Xiao-Ling Tong, Chun-Lin Li, Xin Ding, Zhu-Lin Zhang, Chun-Yan Fang, Duan Tan, Hai Hu, Huai Liu, Fang-Yin Dai

**Affiliations:** 1 State Key Laboratory of Silkworm Genome Biology; Key Laboratory of Sericultural Biology and Genetic Breeding, Ministry of Agriculture and Rural Affairs; College of Biotechnology, Southwest University, Chongqing 400715, China.; 2 College of Plant Protection, Southwest University, Chongqing 400716, China.

**Keywords:** *Bombyx mori*, * Blimp-1*, wing development, transcription regulation

## Abstract

Cys2-His2 zinc finger (C2H2-ZF) proteins represent the most common class of transcription factors. These factors have great potential for the management of developmental progression by regulating the specific spatiotemporal expression of genes. In this study, we cloned one C2H2-ZF protein gene of *Bombyx mori*, *BGIBMGA000319*, that is orthologous to* B-lymphocyte-induced maturation protein-1* (*Blimp-1*); we thus named it as *Bombyx mori Blimp-1* (*BmBlimp-1*). In the silkworm, the *BmBlimp-1* gene is specifically upregulated during day 2 of the pupal to adult stage and is highly expressed in wing discs on day 3 of the pupa. Knockdown of its expression level in the pupal stage results in a crumpled-winged silkworm moth. Using the predicted DNA-binding sequences of *BmBlimp-1* to search the silkworm genome to screen target genes of *BmBlimp-1*, 7049 genes were identified to have at least one binding site of *BmBlimp-1* on their 1 kb upstream and downstream genome regions. Comparisons of those genes with a reported pupal wing disc transcriptome data resulted in 4065 overlapping genes being retrieved. GO enrichment analysis of the overlapping genes showed that most of the genes were enriched in the binding term. Combining functional annotation and real-time quantitative PCR, 15 genes were identified as the candidate target genes of *BmBlimp-1*, including several wing cuticular protein genes, *chitin synthase A*, and wing disc development genes, such as *Wnt1*,* cubitus interruptus* (*ci*) and *engrailed* (*en*). Moreover, the amino acid sequence of the zinc finger motif of *Blimp-1* gene was highly conserved among the 15 insect species. We propose that *BmBlimp-1* is an important regulatory factor in silkworm wing development.

## Introduction

Insects are the most diverse taxonomic group in the world, with more than one million described species. These account for more than half of all known living beings 1,2. Meanwhile, insects are the only invertebrates that have the ability to fly. The flight ability is one of the most important reasons for their diversity. Their wings are not only the core structures for flight, but also play important roles in orientation, protection, communication, and courtship 3. The molecular mechanism of insect wing development has long been an area of special interest.

The larvae of holometabolous insects need to undergo dramatic morphological changes in order to develop into adults. Moreover, their wings, as the flying organs of adults, develop from wing imaginal discs present inside the body of the larvae 4. From *Drosophila melanogaster*, as a standard research organism in insect wing research, a good foundation of knowledge concerning wing imaginal disc development has been collected. Previous research found that two major hormones, 20-hydroxyecdysone (20E) and juvenile hormone, induce and regulate multiple biological processes, including growth, molting and reproduction. JH maintains the larval status, and 20E induces periodic ecdysis and metamorphosis 5. Hormones can regulate the development of wings by initiating or shutting off the expression of relevant genes 6. Previous research has shown that a series of genes are involved in wing early development of *Drosophila*. These genes include transcription factors such as* engrailed* (*en*), *Serum Response Factor* (*SRF*), *cubitus interruptus* (*ci*),* Ultrabithorax* (*Ubx*), *distal-less* (*Dll*), *oculomotor-blind* (*omb*), *apterous* (*ap*), *scalloped* (*sd*)* an*d *vestigial* (*vg*). Moreover, wing development involves secreted proteins such as *Wingless* (*Wg*), *Decapentaplegic* (*Dpp*), *Hedgehog* (*Hh*), and *Fringe* (*Fng*), receptors such as *Notch* (*N*) and *Epidermal growth factor receptor* (*Egfr*), and the ligands *Delta* (*Dl*) and *Serrate* (*Ser*) 7-16. However, many of the factors involved in the molecular mechanisms of wing development remain unknown.

*Drosophila* belongs to Order Diptera, in which the hind wings have degenerated into halteres. However, the Lepidoptera possess more typical insect wings. The morphology of Lepidoptera wings is an emergent representative of other holometabolous insect wings 17. In Lepidoptera, represented by butterflies, the wings have more abundant color schemes than those of flies. Some wing development-related genes also control the wing color pattern in butterflies, for example, the HOX family gene *Ubx* 18. Therefore, the development of Lepidopteran wings is an area of current focus.

The silkworm *Bombyx mori* is an economically important insect that has been domesticated for 5000 years. At present, the silkworm is used as a model species of Lepidoptera for basic research 19,20. In the silkworm, several novel wing development-related genes have been discovered. Twelve wing disc cuticle proteins (WCPs) have been identified (BmWCP1a, BmWCP1b, BmWCP2, BmWCP3, BmWCP4, BmWCP5, BmWCP6, BmWCP7, BmWCP8, BmWCP9, BmWCP10, and BmWCP11) 21. Cuticular protein and chitin are the main components of the insect exoskeleton, which plays an important role in providing stability of the cuticle layer during the wing development process 22. Moreover, dysfunction of adhesion between upper and lower layers of wings causes abnormal wing development. Laminins are a major component of the basal lamina, loss of function of which can produce the blistered wings phenotype in the silkworm 23.

In this study, we found that the *BGIBMGA000319* gene encodes a Cys2-His2 (C2H2) type zinc finger protein orthologous to that of the *B-lymphocyte-induced maturation protein-1* (*Blimp-1*) gene. The *Blimp-1* gene has been identified as a master locus that regulates plasma cell development and immunoglobulin-secreting B-lymphocyte differentiation, and the gene is involved in the differentiation of the myeloid lineage 24-26. Moreover, the expression of the *Blimp-1* gene in primordial germ cells is essential for mouse embryonic development. Loss of function of the gene can lead to embryonic death in mice 27,28. The* Blimp-1* gene participates in determining cell fates in the embryo and plays important roles in multiple hematopoietic lineages. In this article, we report that *Bombyx mori Blimp-1*(*BmBlimp-1*) is specifically upregulated during metamorphosis stages, and that it is highly expressed in the pupal wings on day 3 of the pupal stage. RNA interference (RNAi) resulted in abnormal wings. We thus conclude that the gene participates in the development of the wings.

## Results

### Structural features and phylogenetic analysis of *BmBlimp-1*

**Figure 1:**
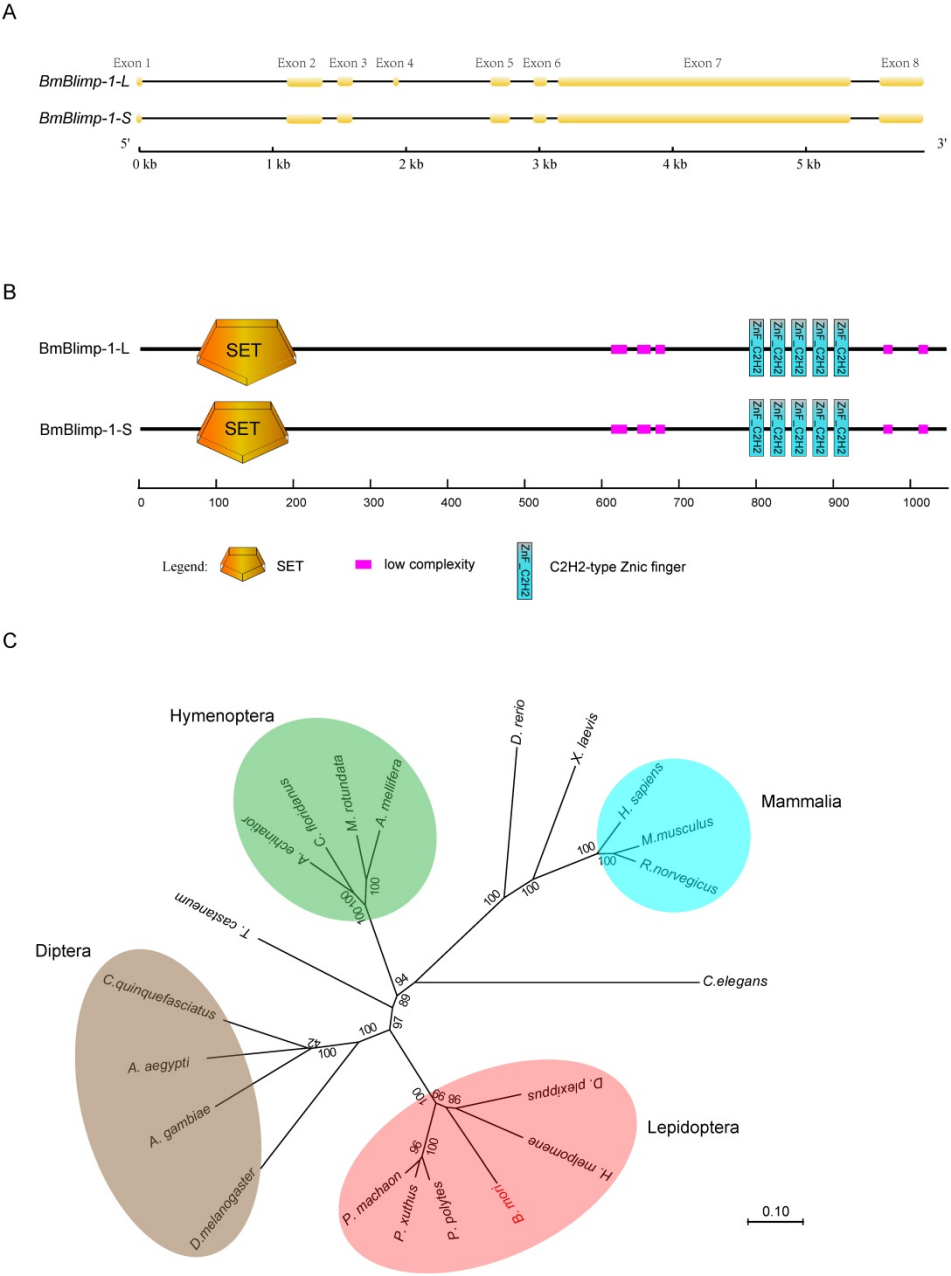
** Structure and phylogenetic trees of the *BmBlimp-1* gene.** (A) The *BmBlimp-1* gene generates two transcripts, a long transcript named *BmBlimp-1-L*, and a shorter transcript named *BmBlimp-1-S*. The yellow squares indicate exons, and the black lines indicate introns. (B) Conservative domain analysis revealed that the two transcripts contained the same number domains; the SET domain of the* BmBlimp-1-S* was 14 amino acids less than that of the* BmBlimp-1-L*. (C) A neighbor-joining phylogenetic tree of the complete nucleotide sequence of *BmBlimp-1* genes among 21 species was constructed using the MUSCLE program and was visualized using the MEGA7 software.

In the silkworm genome database (silkDB, http://www.silkdb.org) 29, a predicted gene* BGIBMGA000319* that is specifically upregulated from the pupal to adult stages was identified by microarray analysis. We speculated that this gene might be involved in pupal development. To explore the function of the *BGIBMGA000319* gene, we first cloned the ORF (open read frame) of this gene from the wild type strain Dazao. Sequence alignment found that the *BGIBMGA000319* gene had alternative splicing; the long transcript was 3213 bp in length and contained eight exons. The short transcript was 3171 bp, which lacked the fourth exon (Fig. 1A). Translation of amino acid (AA) sequences based on the nucleotide sequence indicated that the two transcripts encoded 1070 and 1056 amino acid residues, respectively. Conservative domain analysis of these two predicted proteins was performed using SMART (Simple Modular Architecture Research Tool) software 30. The two proteins shared the same domain that contained one SET (Su(var)3-9, Enhancer-of-zeste and Trithorax) domain and five C2H2-type zinc fingers. The only difference was that the short transcript contained 14 fewer amino acid residues of the SET domain than the long transcript (Fig. 1B). The SET domain can interact with dual-specificity phosphatases (dsPTPases) and contribute to epigenetic effects. The SET domain-containing proteins are involved in histone methylation, which plays an important role in organism growth and development and regulation of gene expression 31. In addition, the C2H2-type zinc finger is considered as a common DNA-binding domain, and genes carrying this domain perform major transcription regulation.

To analyze the homologous genes of *BGIBMGA000319* using its amino acid sequence, we performed a BLAST against the GenBank non-redundant protein sequences (nr) database and found a PR domain zinc finger protein 1(PRDM1) of human that had a sequence homologous to that of the *BGIBMGA000319* gene. The total BLAST score was 511, the E-value was 2e-72, and the percent identity was 66.48%. The PRDM1 protein was also called Blimp-1(B lymphocyte-induced maturation protein 1), and it can regulate the differentiation of mature B cells into plasma cells and can inhibit the expression of genes related to the proliferation of B cells in humans 32. The orthologous gene of *Blimp-1* was found in other Lepidopteran species, such as* Papilio machaon* (*Pm*), *Papilio xuthus* (*Px*), *Danaus plexippus* (*Dp*), *Heliconius melpomene* (*Hm*), and *Papilio polytes* (*Pp*) (Table S1). A phylogenetic tree was constructed based on the nucleotide sequences for *Blimp-1* from 21 species (Fig. 1C). The result showed that the* Blimp-1* gene has only one copy in these species' genomes, and that Lepidoptera, Diptera and Hymenoptera were clustered into a separate group.

### Expression pattern analysis of *BmBlimp-1*

**Figure 2:**
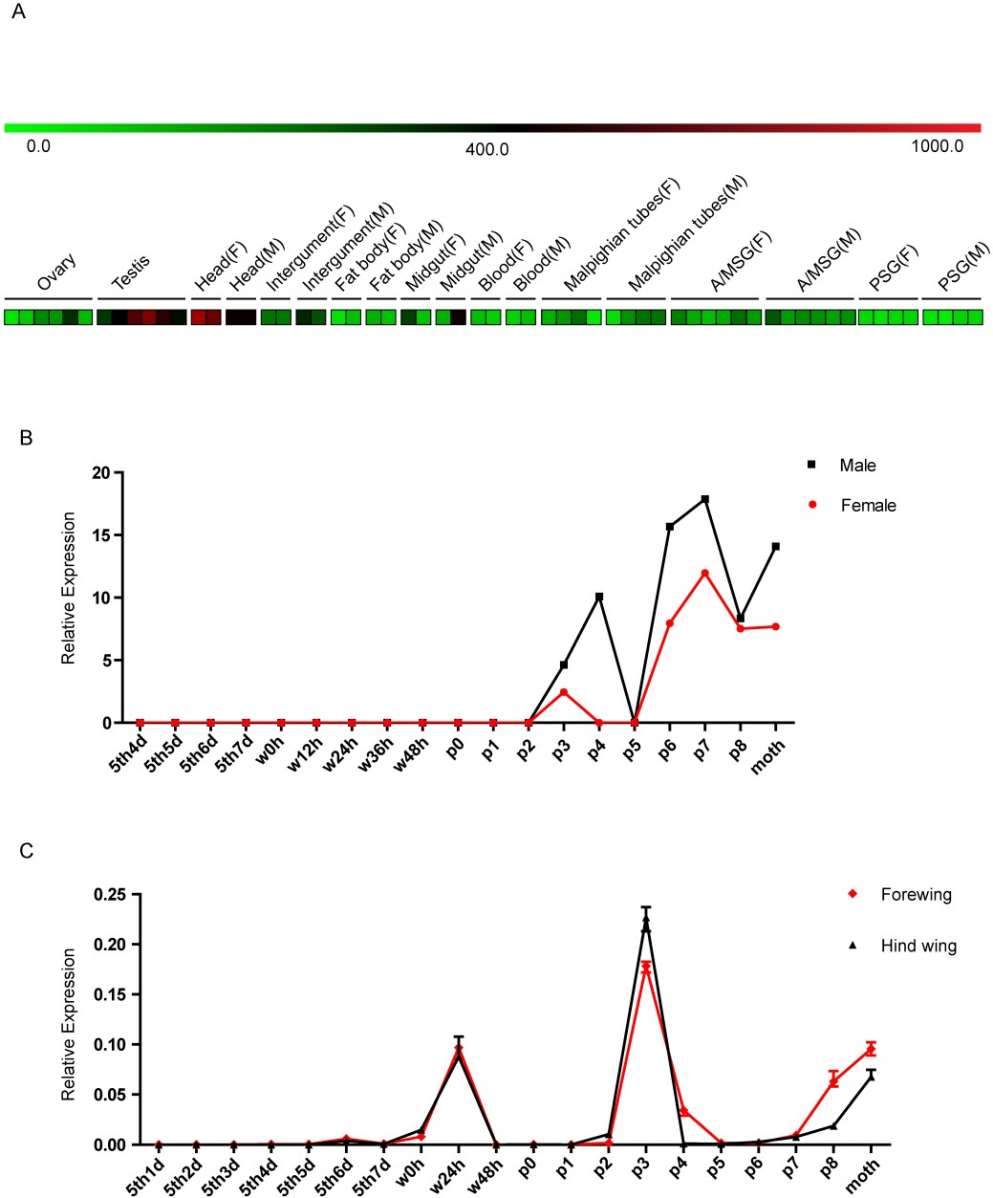
** Expression profiling of the *BmBlimp-1* gene in multiple tissues and developmental stages of the silkworm.** (A) Expression data were downloaded from the BmMDB database. The heat map was constructed using the Mev program. The multiple larval tissues were examined from the third day of the fifth instar, and each tissue had at least two duplications. A value ≥ 400 indicates that the gene was considered as expressed in the tissue. A/MSG: anterior/median silk gland; PSG: posterior silk gland; F: female; M: male. (B) The *BmBlimp-1* gene microarray data during development stages from day 4 of larvae to adult. The developmental time points include day 4 of the fifth instar (5th4d), 5th5d, 5th6d,5th7d, the beginning of the wandering stage for spinning (W0), 12 hours after wandering (W12h), W24h, W36h, W48h (spinning finished), beginning of pupation (P0), one day after pupation (P1), P2, P3, P4, P5, P6, P7, P8 and day 1 of the adult moth. The expression level of the *BmBlimp-1* gene was calculated by comparing gene expression with that in the 5th3d control. (C) The expression pattern of the *BmBlimp-1* gene in wing discs from day 1 of the fifth instar larvae to adult.

Based on sequence and homology analyses of the *BGIBMGA000319* gene, we named it *Bombyx mori Blimp-1* (*BmBlimp-1*). The expression pattern of *BmBlimp-1* was retrieved from the microarray data of the silkworm 33 (Table S2). In multiple tissue chip data of silkworm larvae on day 3 of the fifth instar, the *BmBlimp-1* gene is expressed in low levels in the testis and head, and no expression was detected in eight other tissues (Fig. 2A). In the metamorphosis stage microarray data for the whole body during the later larval to adult stages, the *BmBlimp-1* gene was notably upregulated in pupae and adult stages; expression began on day 2 of the pupa, and the expression peak appeared on the day 7 of the pupa (Fig. 2B). Interestingly, we found an EST (expressed sequence tag) of the *BmBlimp-1* gene, accession number is FS926355, in the EST database of the silkworm in Kaikobase 34; the gene was cloned from the wings of pupal stage moths. This indicates that the *BmBlimp-1* gene was expressed in the wings during the pupal stage. Therefore, expression analysis of the *BmBlimp-1* gene was performed by real-time PCR from the larva to the adult stages in wing discs of the silkworm. The results showed that the expression pattern of forewing and hindwing was consistent, and this gene displayed peak expression on day 3 of the pupa and upregulation at wandering 24 hours, P7, P8 and day 1 of the adult (Fig. 2C). This suggests that the *BmBlimp-1* gene may be related to the development of pupal wings in the silkworm.

### Knockdown of* BmBlimp-1*

**Figure 3:**
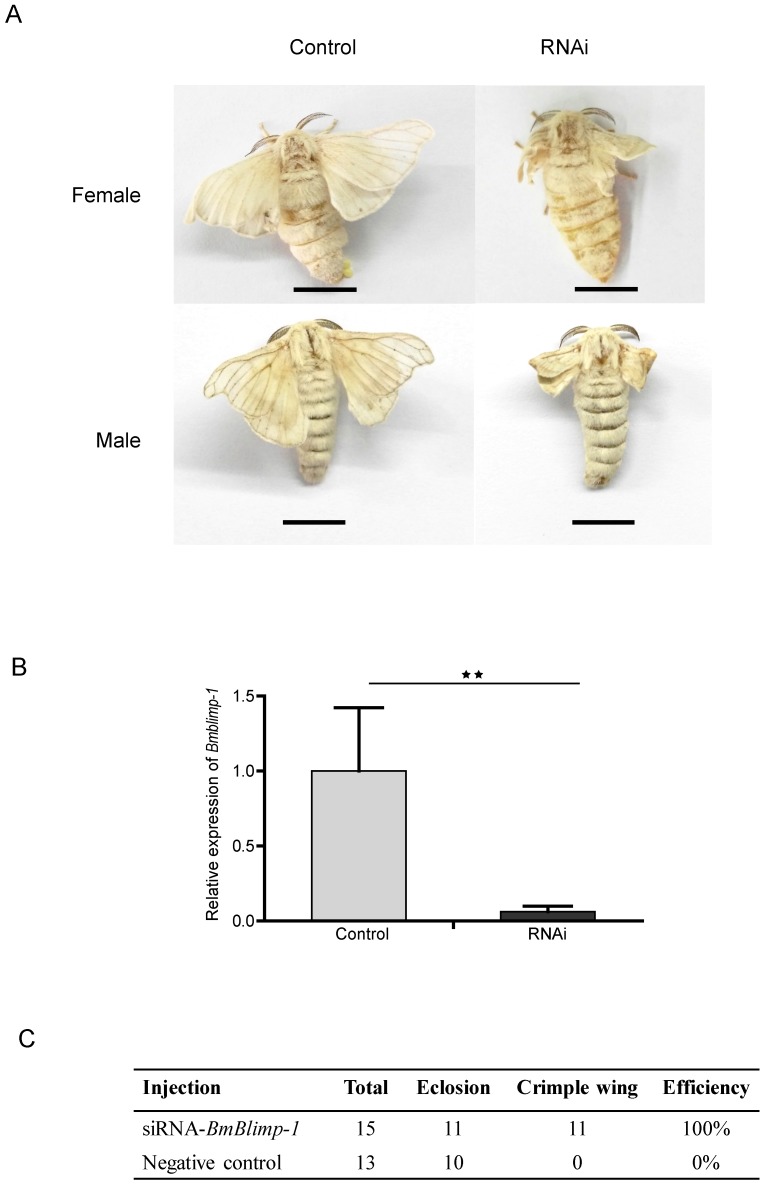
** RNAi of *BmBlimp-1* gene in Silkworm.** (A) Phenotypes of silkworm adults after knockdown of the *BmBlimp-1* gene. The bar indicates 1 centimeter (cm). (B) qRT-PCR of the *BmBlimp-1* gene between control and experimental groups. Statistical analyses were performed using Student's t-test (n = 3). The asterisk indicates statistical significance. (C) Statistics of gene knockdown.

Remarkably, the *BmBlimp-1* gene displayed peak expression in wing discs on day 3 of the pupae, and then was quickly downregulated to almost no expression. This suggests that the predicted transcript factor may perform important regulatory roles in pupae for 3 days. To study the function of the *BmBlimp-1* gene, we synthesized siRNA to knock down the expression level of this gene in the pupae. The individuals of wild type strain Dazao, who pupate for about 36 hours, were selected. Using glass needles, we injected 5 μg siRNA of *BmBlimp-1* into silkworm blood, and the controls were injected with the same quantity of scrambled negative control siRNA. The wings of the experimental group individuals were crumpled and unable to expand, while the control group had normal wings (Fig. 3A). The qRT-PCR showed that the expression level of the *Bmblimp-1* gene was significantly downregulated in the experimental group (Fig. 3B). This indicates that the normal expression of the *BmBlimp-1* gene can maintain the normal development of silkworm wings.

### Downstream target genes analysis of *BmBlimp-1*

**Figure 4:**
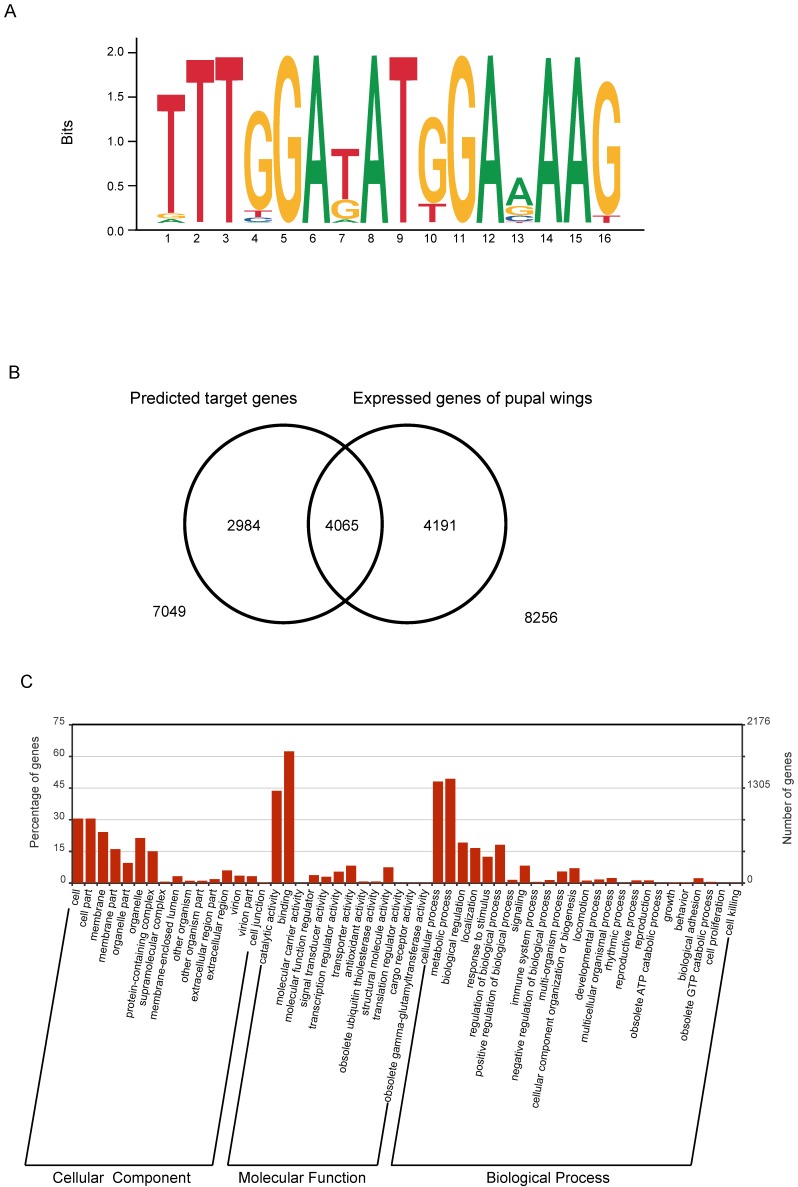
** Predicted target genes of *BmBlimp-1*.** (A) DNA-binding sequences of *BmBlimp-1*. (B) Venn diagram of putative target genes and pupal wing transcriptome data; there are 4055 overlapping genes. (C) GO enrichment analysis of overlapping genes generated by the WEGO program.

The RNA interference experiment showed that knockdown of the expression level of the *BmBlimp-1* gene could cause wing dysplasia in the silkworm. However, *BmBlimp-1* is a C2H2-type zinc finger gene that may function by regulating the transcription of other functional genes. Research has shown that the DNA-binding sequence of the C2H2-ZF motif depends on the amino acid residue sequence of its α-helical component. Considering the first amino acid residue of the α-helical region in the C2H2-ZF domain as position 1, the four positions -1, 2, 3 and 6 are the key amino acids for recognition and binding of DNA via interaction with the hydrogen donors and acceptors exposed in the major groove. In addition, in a group of tandem zinc fingers, the recognition sequences of individual zinc fingers may be modified due to adjacent ZFs. 35,36. Based on empirical calculations of pairwise amino acid-nucleotide interaction energies, the web server http://zf.princeton.edu provides a method for predicting position weight matrices (PWMs) representing DNA-binding specificities for C2H2-ZF proteins 37. Therefore, we predicted the DNA-binding sequence of the *BmBlimp-1* gene on this web site. The predicted DNA-binding sequence was represented as a sequence logo (Fig. 4A). Using FIMO (Find Individual Motif Occurrences) software of the MEME suite 38, we searched the silkworm genome using the predicted DNA-binding sequence, screening the range of one kilobase upstream and downstream of the predicted genes. We identified 7,049 genes that had at least one putative binding site within 1 kb up/downstream and considered each as a possible downstream target gene of *BmBlimp-1*. To investigate the possible downstream genes of *BmBlimp-1* in the pupal wing disc, we compared a reported transcript data of silkworm pupal wings 39. In all, 4065 overlapped genes were found (Fig. 4B), and these genes were used as possible target genes regulated by the *BmBlimp-1* gene during pupal development. The 4065 genes were annotated with Gene Ontology (GO), and 2902 had a GO term. The GO enrichment map was constructed (Fig. 4C); the genes were enriched in binding, metabolic process, cellular process and catalytic activity terms. Furthermore, the 4065 genes were subjected to a BLAST search of the non-redundant sequence database (nr) to functional annotation (Table S3). Multiple reported genes related to wing development were found, including wing cuticular proteins, *Wingless*, and *engrailed*, and those genes were considered as candidate target genes of *BmBlimp-1* for wing development (Table 1).

### Analysis of the expression levels of wing disc development-related genes

**Figure 5:**
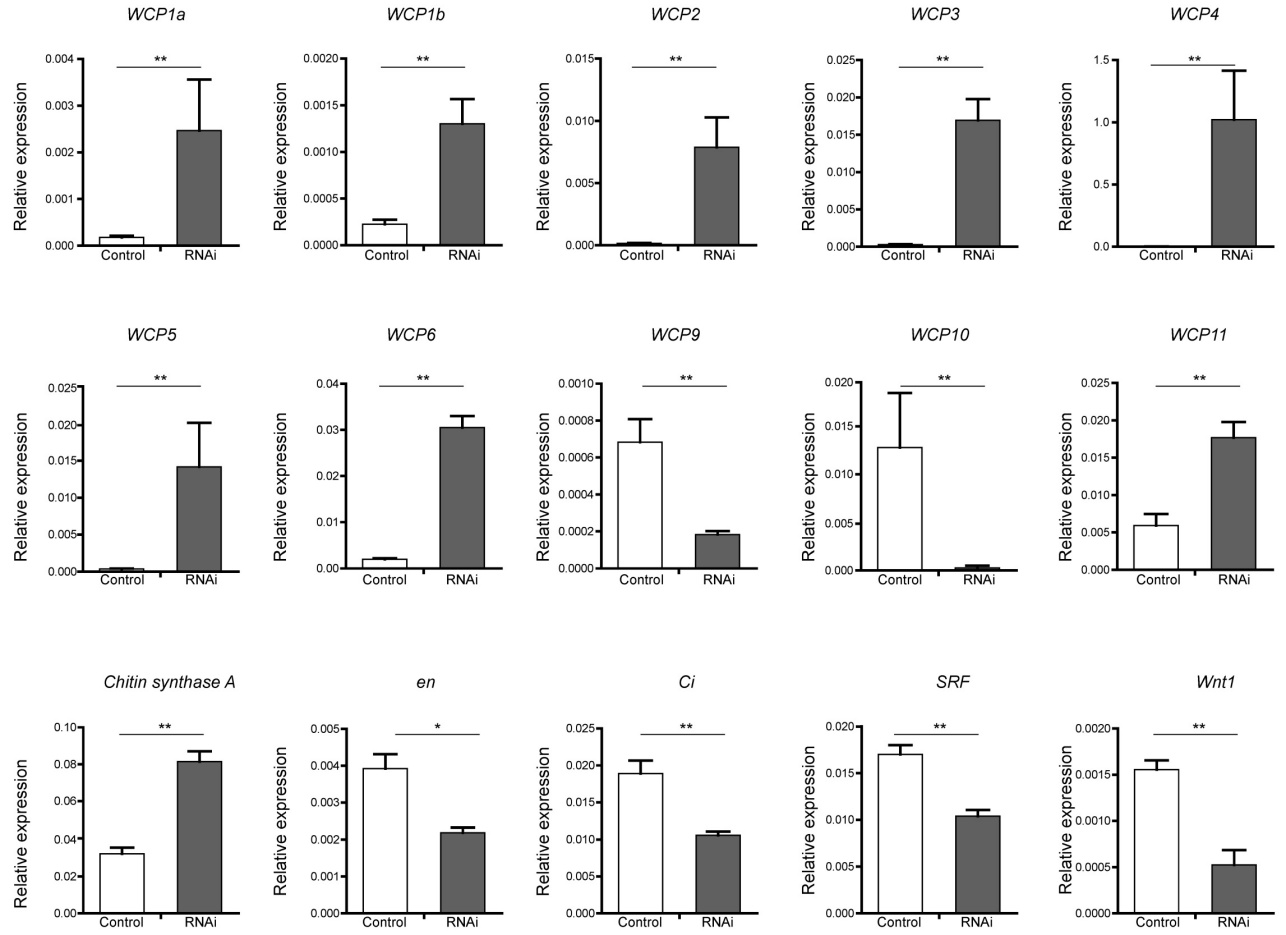
** Expression level of candidate target genes of BmBlimp-1 by qRT-PCR.** The genes, *WCP1a, WCP1b, WCP2, WCP3, WCP4, WPC5, WCP6, WCP9, WCP10, WCP11, chitin synthase A, cubitus interruptus (ci), engrailed (en), Serum Response Factor (SRF) and Wnt1* were significantly differentially expressed in the RNAi treatment group compared with the control group. All data are mean ± S.D (n = 3). Statistical analyses were performed using Student's *t*-test (n = 3). The asterisk indicates statistical significance.

To verify candidate target genes of the BmBlimp-1 protein, we employed real-time fluorescence quantitative PCR to measure the expression levels in RNAi individuals and a control group. *Chitin synthase A* gene and 13 *WCP* genes were investigated. The results showed that eight wing cuticular protein genes (*WCP1a*, *WCP1b*, *WCP2*, *WCP3*, *WCP4*, *WCP5*, *WCP6* and *WCP11*) and the* chitin synthase A* gene were significantly upregulated in the RNAi group (Fig. 5). Meanwhile, *WCP9* and *WCP10* were significantly downregulated. The expression levels of *WCP7* and *WCP8* were very low (not shown in the figure). In addition, eight wing disc development-related genes, *decapentaplegic* (*dpp*), *engrailed* (*en*), *apterous* (*ap*), *Serrate* (*Ser*), *asense* (*ase*), *cubitus interruptus* (*ci*), *Serum Response Factor* (*SRF*) and *Wnt1*, were investigated. Among the factors related to the development of wing discs, we found that *Wnt1*, *en*, *ci* and *SRF* were significantly downregulated (Fig. 5), and that *ap*, *ase* and* Dpp* were not significantly different (data not shown).

### Zinc Fingers Conservation and Binding sites analysis of* Blimp-1*

**Figure 6:**
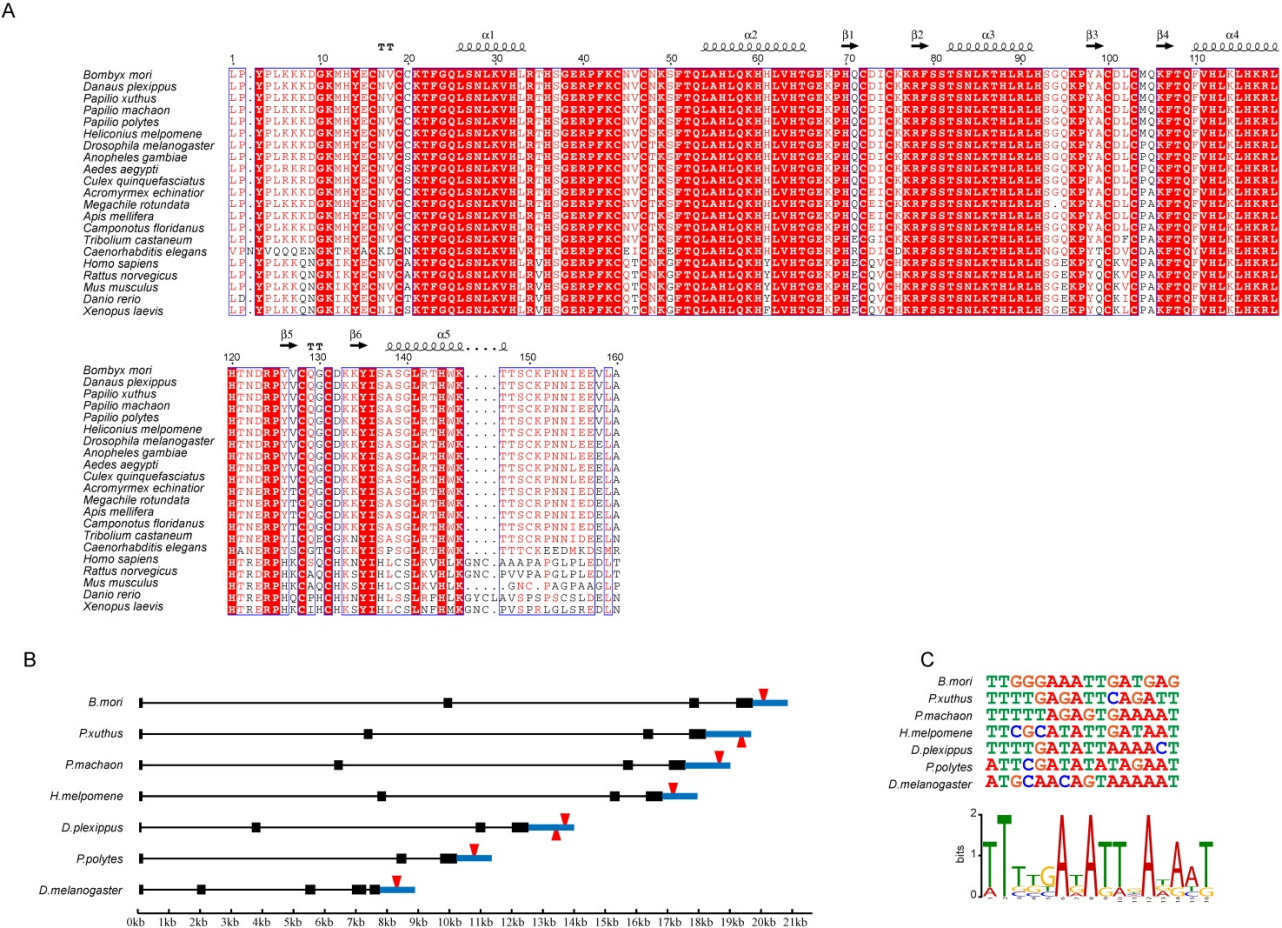
** Conservation Analysis of Zinc Finger Motifs of *BmBlimp-1*.** (A) Amino acids of zinc finger motif analysis among 21 species. (B) *Wnt1* gene structure and DNA-binding site position of *Blimp-1* among seven species. (C) DNA sequences of the binding sites of *Blimp-1* among the seven species, and the sequences logo generated by these DNA-binding sequences.

Genes with important functions tend to be conserved among species. To investigate the degree of conservation of DNA-recognition sequences of the *Blimp-1* gene among species, we compared the AA sequences of zinc finger domains of the *Blimp-1* gene among 21 species. The AA sequences of the ZF domains of the *Blimp-1* gene were aligned (Fig. 6A), illustrating that the sequences of this protein were highly conservative among 15 insects and *Caenorhabditis elegans*. The AA sequences of α-helical regions were completely consistent among 15 insect species, and in *C*. *elegans* only one alanine was changed to proline in the fifth α-helix. In addition, the entire region of the five zinc finger domains shared the same AA residues among the silkworm, *Danaus plexippus* (*Dp*), *Papilio xuthus* (*Px*), *Papilio machaon* (*Pm*) and *Papilio polytes (Pp)*, and there is only one amino acid difference between the silkworm and *Heliconius melpomene* (*Hm*)*,* an asparagine changed to serine. Furthermore, among four vertebrates (*Homo sapenis*, *Mus musculus*,* Rattus norvegicus* and *Danio rerio*), the AA sequences of the α-helical regions are highly conserved. The tandem zinc fingers of *Blimp-1* genes are strongly conserved among species, suggesting the persistence of the highly conserved recognition sequences, and the proteins may regulate some of the same target genes. Therefore, we selected the* Wnt1* gene from the candidates mentioned above; this gene encodes an important secretory protein. The structure of the *Wnt1* gene was analyzed among seven species (*Bm*, *Px*, *Pm*, *Hm*, *Dp*, *Pp* and *Dm*) (Fig. 6B). The *Wnt1* genes in these species have similar structures, and this gene has the longest intron in the silkworm among the species examined. Meanwhile, the DNA-binding sequences of *Blimp-1* were predicted for the seven species. A binding sites analysis of *Blimp-1* to the respective *Wnt1* genes was performed. We compared the binding sites of the Blimp-1 proteins to the* Wnt1* genes in each species. The results showed that a conserved DNA-binding site of *Blimp-1* was present within approximately 1 kb downstream of the *Wnt1* gene in the seven species (Fig. 6C). This suggests that the Blimp-1 protein may bind to and regulate the expression of the *Wnt1* gene in a similar manner among the seven species.

## Discussion

In this study, we found a C2H2 zinc finger protein gene *BGIBMGA000319* that is highly expressed during the silkworm pupal stage. As this gene has sequence homology with the B-lymphocyte-induced maturation protein-1 (*Blimp-1*) gene in vertebrates, we named it *BmBlimp-1*. RNAi experiments found that knockdown of the expression level of this gene at the peak expression stage causes abnormal wing development in the silkworm. Using DNA-binding motif sequence prediction, silkworm whole-genome analysis, and comparative transcriptome data of wing discs of silkworm pupae, we screened for genes related to wing development and detected genes with significant changes in expression levels in the knockdown group. The candidate target genes of *BmBlimp-1* were identified, and these genes included *Wnt1*, *engrailed* (*en*), *asense* (*ase*), *cubitus interruptus* (*ci*), *Serum Response Factor* (*SRF*), wing cuticular protein genes (*WCP1a*, *WCP1b*, *WCP2*, *WCP3*, *WCP4*, *WCP5*, *WCP6*,* WCP9*, *WCP10* and *WCP11*) and *chitin synthase A*. Furthermore, we found that the zinc finger motif of the *Blimp-1* gene is highly conserved among multiple insect species, which suggests that this gene may perform homologous and important functions among species.

The *Blimp-1* gene participates in determining cell fate and plays important roles in multiple hematopoietic lineages in vertebrates. In *Drosophila*, the *Blimp-1* gene is induced directly by 20-hydroxyecdysone (20E), and its gene product exists during the high-ecdysteroid phase. This gene can bind to and regulate the transcription of* ftz-f1*, a nuclear receptor transcription factor that plays an important role in molting and metamorphosis. Knockdown of *Blimp-1* can lead to pre-pupal lethality, while prolonging its expression results in delay of pupation 40. In embryogenesis, *Blimp-1* is critically required for the development and maturation of the tracheal system 41. In the silkworm, *BmBlimp-1* is also upregulated during the pupal stage. This suggests that a similar mechanism is shared between the silkworm and the fruit fly, in which *BmBlimp-1* may be directly induced by 20E and may participate in pupal development. According to the ecdysterone titer level of *Bombyx mori* 42, this gene is upregulated in the late pupae and moth stages, while the ecdysone titer is at a low level during this period (Fig. S1). Therefore, the expression of *Blimp-1* in the silkworm may be activated by other factors.

In the GO analysis of possible direct target genes of the BmBlimp-1 protein, we found that genes were most enriched in the binding term. This term had 63 genes; these genes were mainly predicted to be transcription factors. This suggests that the* BmBlimp-1* gene acts as an upstream factor in a regulatory network for pupal wing development. In the qRT-PCR verification of candidate target genes, although no binding sites of the BmBlimp-1 protein within 1 kb upstream and downstream of *WCP1a*, *WCP4*, *WCP5* and *WCP6* genes were found, their expression levels changed significantly. There are several possibilities. First, those genes, except for* WCP6*, are serially arranged on chromosome 22, and thus, they may share the same cis-regulatory element. Second, we only retrieved the 1 kb upstream and downstream regulatory sites and neglected the longer positions and introns. Third, it is possible that the protein may indirectly regulate the expression levels of these genes by regulating other TFs. In addition, several wing cuticular protein genes were mainly expressed in the wing discs of early pupae, and the expression level declined by day 3 of the pupa 43. Knocking down the expression of the *BmBlimp-1* gene caused several *WCP* genes to be remarkably upregulated, and the knockdown also resulted in significant downregulation of the *WCP9* and *WCP10* genes. This implies that the* BmBlimp-1* gene may act to activate and inhibit expression as a dual-function regulator. Cuticular proteins and chitin are the major components of the epidermis of the exoskeleton and wings. Many cuticular proteins have a chitin-binding domain, and diverse groups of these complexes are orderly cross-linked to form a stable structure. When one or more of the cuticular proteins is disordered, this can lead to structural changes or tissue abnormalities 44,45. The changes in the expression of wing cuticular protein genes and *chitin synthases A* in the RNAi group may result in an abnormal wing. Meanwhile, the wing disc pattern genes such as *Wnt1*, *en*, *ci* and* SRF* were significantly down-regulated in the RNAi group. These genes play important roles in wing disc development in the embryonic stages, as shown in previous wing studies with flies. However, these genes are also expressed in the pupal wings of the silkworm, and as candidate target genes of *BmBlimp-1*, they were markedly down-regulated in RNAi individuals. We suggest that these genes may be directly regulated by *BmBlimp-1*, and that they participate in pupal wing development.

In multiple species aligning, we found that the amino acid sequence of *Blimp-1* was highly conserved; in 15 insect species examined, the amino acid residues of the zinc finger motif were very similar. This indicated the existence of similar binding sites of *Blimp-1* in those species. The discovery of conserved binding sites downstream of the *Wnt1* gene in seven insects also fits this hypothesis.

This study has identified the function of the *BmBlimp-1* gene in pupal wing development of the silkworm. Combined with the analysis of direct target genes, we believe that the *BmBlimp-1* gene may regulate the expression of several *WCP* genes along with the wing disc development-related genes *Wnt1*, *en*, *ci* and *SRF* to participate in pupal wing development in the silkworm. The results serve as motivation for studying the conservative C2H2 zinc finger protein TFs, which are involved in the wing development of the silkworm and other species of Lepidoptera. The candidate target genes analyzed by our screening can provide references for further functional research.

## Materials and Methods

### Silkworm strain and tissue collection

The wild-type strain Dazao was obtained from the Silkworm Gene Bank of Southwest University, Chongqing, China. Silkworms were reared on mulberry leaves at 25°C in 75% relative humidity under stable conditions (12-h light: 12-h dark). The wing discs were removed using 0.7% normal saline solution and then stored at -80°C until RNA extraction was performed.

### Gene cloning and RT-PCR

Total RNA was isolated from the silkworm Dazao strain and wing discs using TRIzol® reagent (Invitrogen, Carlsbad, CA, USA) and E.Z.N.A.® MicroElute Total RNA Kit (Omega Bio-tek, Norcross, GA, USA) according to the manufacturer's protocol. The primers that were used for qRT-PCR are listed in Supplementary Table S4. The qRT-PCR was performed using a CFX96™ Real-Time PCR Detection System (Bio-Rad, Hercules, CA) with SYBR Green qRT-PCR Mix (Bio-Rad). The cycling parameters were as follows: 95°C for 3 min followed by 40 cycles at 95°C for 10 s and annealing for 30 s. The relative expression levels were analyzed using the classical R= 2^-ΔΔCt^ method. The gene for ribosomal protein L3 (rpL3) of the silkworm was used as an internal control to normalize for equal sample loading.

### Phylogenetic analysis

To determine whether *BGIBMGA000319* (*BmBlimp-1*) orthologues existed in species other than *B. mori*, we performed a phylogenetic analysis using genes included in the Blastp hits with E-values ≤10^-5^ in the NCBI nr database (http://www.ncbi.nlm.nih.gov/BLAST/), with *Drosophila melanogaster* (http://www.flybase.org/) 46, the Monarch genome (http://monarchbase.umassmed.edu/) 47, the *Heliconius melpomene* genome (http://butterflygenome.org/) 48, and *Tribolium castanenum* (http://beetlebase.org/) 49. Alignment was generated from a multiple sequence alignment of DNA sequences by MUSCLE 50 and was used to construct nucleotide alignments. The phylogenetic tree was constructed using the neighbor joining method using the MEGA7 program 51. The confidence levels for various phylogenetic lineages were assessed by bootstrap analysis (1,000 replicates).

### siRNA for gene knockdown

The siRNAs for *BmBlimp-1* were designed to target 5'-GCCAAAUACACCGACUGAATT-3' for gene knockdown, and the scramble negative control 5'-AGAACCAGAUAACCGAUCCTT-3' (Genepharma, China) was used as a negative control. Five microliters of siRNA 1μg/μl was injected from the chest spiracle into the hemolymph at 36 hours after pupation. After injection, the individuals were maintained at 25°C in 75% humidity and 12 hours light and 12 hours dark until eclosion. Quantitative RT-PCR was employed to measure the mRNA levels of target genes of samples that were obtained at day 4 of the pupa.

### DNA-Binding Site Prediction and Target Gene Screening

To obtain the DNA-binding site position weight matrices for the BmBlimp-1 protein, an online de-novo prediction website (http://zf.princeton.edu) was used, and the linear expanded predicted method was chosen 37. The FIMO program of the MEME software suite was used to search for binding sites in the silkworm genome 52. Screening the range of 1 kb upstream and downstream of genes was done using an R-script. Comparing the screened genes with known pupal wing transcriptome data yielded possible target gene sets. The GO analysis results of those possible target genes were plotted using the WEGO (Web Gene Ontology Annotation Plot) program (http://wego.genomics.org.cn) 53.

### Conservation Analysis of the Zinc Finger Structure of Multi-Species *Blimp-1*

Alignment of the amino acid sequences of the zinc finger structures of 21 species using the MUSCLE program followed by the Espript 3.0 (Easy Sequencing in PostScript) program 54 was used to render sequence similarities and secondary structure information from the aligned sequences. The sequences of *Wnt1* genes of seven insect species were downloaded from NCBI. The prediction of *Blimp-1* binding sites of *Wnt1* genes in different species followed the method described above. The sequence logo of binding sites downstream of *Wnt1* in seven insects was generated by the MEME program.

## Supplementary Material

Supplementary figures and tables S1, S2, S4.

Table S3.

## Figures and Tables

**Table 1:** Candidate target genes of *BmBlimp-1* related to wing development

Gene	SilkDB ID	Sequence location
*WCP1b*	*BGIBMGA000429*	nscaf1681: 3265316-3270930 (+)
*WCP2*	*BGIBMGA000265*	nscaf1681: 3249518-3250498 (-)
*WCP3*	*BGIBMGA000269*	nscaf1681:3231459-3232623 (-)
*WCP9*	*BGIBMGA000325*	nscaf1681: 697463-702380 (-)
*WCP10*	*BGIBMGA001444*	nscaf2136: 2851152-2860721(-)
*WCP11*	*BGIBMGA000324*	nscaf1681: 710443-712032 (-)
*chitin synthase A*	*BGIBMGA002963*	nscaf2589: 6004788-6016095 (-)
*decapentaplegic* (*dpp*)	*BGIBMGA010384*	nscaf2993: 6692859-6696343 (-)
*engrailed* (*en*)	*BGIBMGA009644*	nscaf2964: 4195216-4196156 (-)
*wingless* (wg)	*BGIBMGA006146*	nscaf2847: 4290086-4300264 (+)
*apterous* (*ap*)	*BGIBMGA002127*	nscaf2210: 3499610-3502050 (+)
*Serrate* (*Ser*)	*BGIBMGA000783*	nscaf1705: 3574-11539 (-)
*asense* (*ase*)	*BGIBMGA001002*	nscaf1898: 5940257-5941471 (-)
*cubitus interruptus* (*ci*)	*BGIBMGA004545*	nscaf2800: 2013357-2027060 (-)
*Serum Response Factor* (*SRF*)	*BGIBMGA001384*	nscaf2110: 8727-9565 (-)
	*BGIBMGA001382*	nscaf2108: 36519-37205 (+)
