# Supplementary material for: BmBlimp-1 gene encoding a C2H2 zinc finger protein is required for wing development in the silkworm Bombyx mori
Source: Int J Biol Sci. 2019 Oct 12;15(12):2664–75. doi: 10.7150/ijbs.34743 (PMC6854374; doi:10.7150/ijbs.34743)
Supplement: Supplementary file 1 — Supplementary figures and tables S1, S2, S4. [file ijbsv15p2664s1.pdf]

Supplementary materials

Table S1. The nucleotide sequences of *BmBilmp-1* of the silkworm and the ORF of *Blimp-1* of other 20 species used in constructing the phylogenetic tree.

>*BmBlimp-1-L*

ATGAGACTCGCAAATAGCGAAGAAGTACCAAGTACAACAGTGGTGGGCCGCAGCAACAACGCGAATAGCCCTGAAAGACTCGGCCGGCTTACA
GCAGTACCAGACTCAGGAGCCTACGATGTAACACGCATGCGTGAGGAAGAATTTGAAAGGCTAACTGTGTACATAGTCCCCGACGTACCATGCG
AAAGGGGAACATTAAACAGAGCTGAAAAAACACTCCCGCGCAGTCTGACTTTACGACCATCTGCAGTGCTATCGACGCCAAATACACCGACTG
AAGGAGTATGGAGCATTGGTGTCATACCACGGGGAACGCGCTTCGGGCCGTTTGAGGGAACTAGAACCCCAAATAAACCTAACGACAAAATTTC
TTGGCGTTATTACTGGAGAGTACAGGAAACTGTCAGACATCATACATACGGAAATATTAAGATATTCATAGATAGTGATTATTATTACCTGGATGGG
TCGGATACAAGCGTCGCCAATTGGATGCGATACGTCGCTTCCGCGTATTCGCTCTCAGTAATGAACCTCGTTGCTTGTCAGCACCAAGAACACAT
ATATTTCTACACTGTCAGGGACATCATGCCCAATGAAGAGCTAATGGTGTGGTATTGTAAAGATTTTGCAACGAGATTAGGATATGACATCGATCC
AGAAAGAGCTACATACTCTATATGTAAAGAAGAAACTATTAAGAAAGCATACGGCCTGCCACCTGCGCCCGTACATCCCGAAATCGCTTTTAATC
ATATGAAATACGTACTTGGCTTGACCAATGCTAAAGAACTACTAGAAACTGAAATACCAAGTCGCAGGCGAAAACGTGAAAATAGTGATAAACAT
GACAATCAAAACGTTCTTATCAGAGAAGATCCACGTTGCATTGCTAAAAATGAGAGAACGATAGTTCGGACTGACATTTCTACAAATATAGTCCA
CAATAGTGACACACCTATTATTAACGAACAGTCTGCATCTGGAAGTCTACTTCCAGTTAAAATGGCCCATAGAGAAAACCCAACGCTAATAATAC
ACCAGCATAAAGAAAGCACTTCTCAATTGGTAATACACCAACTAGAAAACCCGAGACTTCAAAGTAACCGATCACCCATTCAAAGTCACCTACG
TGATGTTAGAGTTTCATCACCTCTGACACACCATCACGATAAACCTGGAAACAAGTCACCTATGTTGACTCACTGTGGTGGACCATCATACGAAC
ATCAACTAACTCCCAATGATGGATCGGTGCGGTCTGACGAAGGCTATCATAGCAACGGATACCATGATGAATTCACACCACCGGAAGATTCAAGT
GATTCCGATGTTGAAAACTACGTTCTCGACTGTTCCAATAAGACGAATGAACCAAAAGAGACCGTCATAGTACAAAAAATGGAACAAGACATGC
ATCGTAATGAATACAGAAAAGTGAAAATAAAAATGCCAAGAGCATATCAATATCAAAACCATAAAGACATGGACTGTGAAAGTGAAACTGGGTT
GGTTATTGCTGAAGATGTTCCAGACAATATGCCACAAAACTTGTCACCACCGAGAAGAGATCCAGCGACTTCGACTGTTATCGTATTGGAGTCTT
CACAAAATACTGTTGTGCCCATTAACAAGCCTTATTATGAAGATGGTCCGCTTTCTCCTTCTCCACAACCAGCTTTTATGAGGTATTCGCCCCCTG
ATACACGTATTCTAGAAACTATACTCACAGGAAACAGAATAGACACCAATAACAATGACCCCACTCGTCGTCAACCCAATGCAACCCCGCCACCT
TCTTCGCCAACGGAAATGGCCTACTCATACAAGAAAAGCCAAAGATATGGTAACGCATGCAGTCCCGACTCAAGCTCTAATTTAACGCCCTTGCC
ATCCTTACTGCCACTGCCTCAACAATTACCTACTCCAAATGCTGTTCCAGTTTACTCTGCATCACCGCCTCACTCGATACCCCACACAACGGAAAT
ACGTGAAATCAGAGAAATCAGAGAAATTCGGGAAACTCGTTACGAGAGTCATTATCCCAATTATTCTTACAATTTGTATTCCCCGCCGAATTCGAC
TATCATAACTCAGCTAGGATCACCGCCGAATACGTATTCACCTACGGTCACGTCAACACATCAGTACGATAGATCACAAAATGGACAGAGTAATC
ATCACTATCCGTCATCATCAACAAGTGTCATTACACTTAAGAATTGCTCACAATTAAGTTTGATTCAAAATGGAAATGTCGGAGGACAAGTACTTC
CGCCGCATGGGAGCGTGAGTCCCGATGGTAGCTGTGGACTCATGATGTCACCGATTAGTCCTAATTCTCAAGCGTCTCGGGGGTACAGGAGCTTA
CCTTATCCTCTAAAAAAGAAGGACGGAAAAATGCATTATGAATGTAACGTTTGTTGTAAGACTTTTGGTCAATTGTCGAATCTCAAAGTCCATTTA
AGGACACATTCCGGAGAAAGACCGTTCAAATGCAATGTATGTAATAAATCGTTTACTCAACTAGCTCATTTACAAAAACATCACTTAGTGCATACT
GGAGAAAAGCCACATCAATGTGATATTTGCAAGAAACGTTTTTCTTCTACATCGAATTTAAAAACACATTTACGTCTCCATTCAGGACAAAAACC
TTATGCTTGTGATTTATGTATGCAAAAGTTTACACAATTCGTTCATTTGAAACTACACAAAAGACTTCACACTAACGATCGTCCGTACGTCTGCCA
AGGTTGTGACAAAAAATATATAAGCGCATCTGGTTTGCGAACACATTGGAAAACAACGAGCTGTAAACCAAATAATATTGAAGAAGTATTGGCCA
TCACAAACGCAGCTAACACTGAATGCATCGGTAACGTCGACAAAGACTGTCACGAAAGAGAGGGGCACGATGCTTACGAGGTGCACTCTCCAT
CACAAGGAGGGCTGGTGGGAGCCGGTGGGGCGCGTGTCGCAATGTCACACAGTGAAGCCATGCTGGGAGGGCACGCGCACACTACCAGACCG
CACTTAGCGTCAGGACACGGACAATTGTCGCCAGCCCACGGAACTCAATACCGTCCCTTAGCACCTCCACCACCCCCTGATCATGATCAACAGC
AGCCGCATTATAATTCAGCCGGAACCGAGCCCAGGCCTAGCGTTATAGAATCCAGCCAGCCACTCATCATAGAATGCACCTGA

> *BmBlimp-1-S*

ATGAGACTCGCAAATAGCGAAGAAGTACCAAGTACAACAGTGGTGGGCCGCAGCAACAACGCGAATAGCCCTGAAAGACTCGGCCGGCTTACA
GCAGTACCAGACTCAGGAGCCTACGATGTAACACGCATGCGTGAGGAAGAATTTGAAAGGCTAACTGTGTACATAGTCCCCGACGTACCATGCG
AAAGGGGAACATTAAACAGAGCTGAAAAAACACTCCCGCGCAGTCTGACTTTACGACCATCTGCAGTGCTATCGACGCCAAATACACCGACTG
AAGGAGTATGGAGCATTGGTGTCATACCACGGGGAACGCGCTTCGGGCCGTTTGAGGGAACTAGAACCCCAAATAAACCTAACGACAAAATTTC
TTGGCGTTATTACTGGAGAATATTCATAGATAGTGATTATTATTACCTGGATGGGTCGGATACAAGCGTCGCCAATTGGATGCGATACGTCGCTTCC
GCGTATTCGCTCTCAGTAATGAACCTCGTTGCTTGTCAGCACCAAGAACACATATATTTCTACACTGTCAGGGACATCATGCCCAATGAAGAGCTA
ATGGTGTGGTATTGTAAAGATTTTGCAACGAGATTAGGATATGACATCGATCCAGAAAGAGCTACATACTCTATATGTAAAGAAGAAACTATTAAG
AAAGCATACGGCCTGCCACCTGCGCCCGTACATCCCGAAATCGCTTTTAATCATATGAAATACGTACTTGGCTTGACCAATGCTAAAGAACTACTA
GAAACTGAAATACCAAGTCGCAGGCGAAAACGTGAAAATAGTGATAAACATGACAATCAAAACGTTCTTATCAGAGAAGATCCACGTTGCATTG
CTAAAAATGAGAGAACGATAGTTCGGACTGACATTTCTACAAATATAGTCCACAATAGTGACACACCTATTATTAACGAACAGTCTGCATCTGGAA
GTCTACTTCCAGTTAAAATGGCCCATAGAGAAAACCCAACGCTAATAATACACCAGCATAAAGAAAGCACTTCTCAATTGGTAATACACCAACTA
GAAAACCCGAGACTTCAAAGTAACCGATCACCCATTCAAAGTCACCTACGTGATGTTAGAGTTTCATCACCTCTGACACACCATCACGATAAACC

TGGAAACAAGTCACCTATGTTGACTCACTGTGGTGGACCATCATACGAACATCAACTAACTCCCAATGATGGATCGGTGCGGTCTGACGAAGGCT
ATCATAGCAACGGATACCATGATGAATTCACACCACCGGAAGATTCAAGTGATTCCGATGTTGAAAACTACGTTCTCGACTGTTCCAATAAGACG
AATGAACCAAAAGAGACCGTCATAGTACAAAAAATGGAACAAGACATGCATCGTAATGAATACAGAAAAGTGAAAATAAAAATGCCAAGAGCA
TATCAATATCAAAACCATAAAGACATGGACTGTGAAAGTGAAACTGGGTTGGTTATTGCTGAAGATGTTCCAGACAATATGCCACAAAACTTGTC
ACCACCGAGAAGAGATCCAGCGACTTCGACTGTTATCGTATTGGAGTCTTCACAAAATACTGTTGTGCCCATTAACAAGCCTTATTATGAAGATG
GTCCGCTTTCTCCTTCTCCACAACCAGCTTTTATGAGGTATTCGCCCCCTGATACACGTATTCTAGAAACTATACTCACAGGAAACAGAATAGACA
CCAATAACAATGACCCCACTCGTCGTCAACCCAATGCAACCCCGCCACCTTCTTCGCCAACGGAAATGGCCTACTCATACAAGAAAAGCCAAAG
ATATGGTAACGCATGCAGTCCCGACTCAAGCTCTAATTTAACGCCCTTGCCATCCTTACTGCCACTGCCTCAACAATTACCTACTCCAAATGCTGT
TCCAGTTTACTCTGCATCACCGCCTCACTCGATACCCCACACAACGGAAATACGTGAAATCAGAGAAATCAGAGAAATTCGGGAAACTCGTTAC
GAGAGTCATTATCCCAATTATTCTTACAATTTGTATTCCCCGCCGAATTCGACTATCATAACTCAGCTAGGATCACCGCCGAATACGTATTCACCTAC
GGTCACGTCAACACATCAGTACGATAGATCACAAAATGGACAGAGTAATCATCACTATCCGTCATCATCAACAAGTGTCATTACACTTAAGAATTG
CTCACAATTAAGTTTGATTCAAAATGGAAATGTCGGAGGACAAGTACTTCCGCCGCATGGGAGCGTGAGTCCCGATGGTAGCTGTGGACTCATG
ATGTCACCGATTAGTCCTAATTCTCAAGCGTCTCGGGGGTACAGGAGCTTACCTTATCCTCTAAAAAAGAAGGACGGAAAAATGCATTATGAATG
TAACGTTTGTTGTAAGACTTTTGGTCAATTGTCGAATCTCAAAGTCCATTTAAGGACACATTCCGGAGAAAGACCGTTCAAATGCAATGTATGTA
ATAAATCGTTTACTCAACTAGCTCATTTACAAAAACATCACTTAGTGCATACTGGAGAAAAGCCACATCAATGTGATATTTGCAAGAAACGTTTTT
CTTCTACATCGAATTTAAAAACACATTTACGTCTCCATTCAGGACAAAAACCTTATGCTTGTGATTTATGTATGCAAAAGTTTACACAATTCGTTCA
TTTGAAACTACACAAAAGACTTCACACTAACGATCGTCCGTACGTCTGCCAAGGTTGTGACAAAAAATATATAAGCGCATCTGGTTTGCGAACAC
ATTGGAAAACAACGAGCTGTAAACCAAATAATATTGAAGAAGTATTGGCCATCACAAACGCAGCTAACACTGAATGCATCGGTAACGTCGACAA
AGACTGTCACGAAAGAGAGGGGCACGATGCTTACGAGGTGCACTCTCCATCACAAGGAGGGCTGGTGGGAGCCGGTGGGGCGCGTGTCGCAA
TGTCACACAGTGAAGCCATGCTGGGAGGGCACGCGCACACTACCAGACCGCACTTAGCGTCAGGACACGGACAATTGTCGCCAGCCCACGGAA
CTCAATACCGTCCCTTAGCACCTCCACCACCCCCTGATCATGATCAACAGCAGCCGCATTATAATTCAGCCGGAACCGAGCCCAGGCCTAGCGTT
ATAGAATCCAGCCAGCCACTCATCATAGAATGCACCTGA

>*Danaus_plexippus* DPOGS207967

ATGCGTGAAGAAGAGTTTGAAAGGCTCACAGTATACATAGTACCCGACGTGCCATGTGAACGAGGAGTGACAAACAGAGCAGAGAGAACGCTC
CCTCGGAGTTTGGCACTGAGGCCATCTGCAGTTCTATCTACACCCAACACACCGACGGAAGGAGTCTGGAGTAAGGGAGTTATACCCCGCGGTA
CGAGATTCGGACCATTTGAAGGCACACGAACACCTAATAAACCAAATGACAAAGTTTCGTGGCGATATTATTGGAGGGTACAAGAAACTGTCAG
ACATCATACTTATGGAAATATTAAGATATTCAAAGATAGTGACTATTATTACCTTGATGGATCGGACACCAGCGTCGCTAACTGGATGAGATATGTC
GCCTCAGCATATTCCCTTTCCGTCATGAATCTCGTTGCTTGTCAACATCAAGAACACATTTACTTTTACACAATCAGAGACATTATGCCTAATGAAG
AACTTATGGTATGGTACTGTAAGGATTTTGCTACAAGGCTTGGTTATGACATTGATCCTGAAAGAGCAACTTATTCAATATGCAAGGAAGAAACAA
TAAAAAAGGCGTATGGATTGCCACCTGCCCCTGTACATCCTGAGATTGCTTTTAACCACATGAAATATGTTCTCGGTCTGACAAACGCTAAAGATC
TTTTAGAGGCAGAAATTCCGAGCAGGAGAAGAAAAAGAGAAGCGAGTGATAAATCACTACACGAAGATCAAATCATGTTACCTCGTGAAGATC
CCAGAAATGTTATAAAAAATGAGAGATCTATCGTAAGGTCAGATATCGCCACAAATTTCATACACCCGAAATCTGAGAGCCCAAATCATAACGAA
CATCACTCACTATCACCTAGCAACTTAATGCAGCCTCCATCACCGGTTAAAATGCATAGAGATAATCAAACGTTAATAATTCATCAGCACAAAGAA
AGCACATCGCATGTTGTTATACATCAACTTGATAATTCAAGATCACATAGCAATCGATCCCCTGCTCAAATTCAACATACGAGGGATATTAGAGGA
CCATCTCCGTTATCACAAAATAATCAAGACAAACTTGGAAGTAAATCTCCCAGTTTTCCTCATTGTGTACCATCACATTATGAGCACCAACTGACA
CCTACTGATGGTTCAGTTCGGTCAGATGAAGGTTATCATAGCAATGGATATCACGATGAATTTACTCCCCCAGAAGACTCGAGTGACTCCGATGTC
GAAAATTATGTACTAGACTGTTCAAATAAAACAAATGAACCAAAAGAAACTGTTATTGTTCAAAAAATTGATCAGGATATGCATAGAAATGAATA
CAGAAAAGTTAAAATAAAAATGCCCAGAGCTTATCAATACCAAAATCACAAAGATATGGATTGTGAAAGTGAAAAAGAAATGATTATAGCCGAG
GACGTTATGCACACCTCGCCTCAAAATTTATCTCCAACCCGAAGAGATCCTGCTAGTTCTACGGTTATCGTTCTAGAGTCCTCTCAAAACACAGTA
GTACCTTTAAATAAGCCTTACTATGAAGACGGTACATTATCGCCTTCACCACAGCCAGCTTTTATGAGATACTCGCCCCCAGATACAAGAATACTT
GAAACAATACTGACCGGAAATAGGATTGATACAAATAATAATGACCCAAATAGAAGACAACCAAATGCTACTCCACCACCATCATCGCCCACAGA
GATGGCGTACTCCTACAAGAAAAGTCAGAGATATGGCAATGCTTGCAGCCCTGATTCCAGTTCAAACCTAACGCCTCTACCATCTTTATTGCCGTT
ACCACAACAGTTACCTACACCTAATTCAGTTTCGGTTTATTCTTCTTCACCTCCGCATACTATTCCTCATACTACAGAAATTCGTGAAATACGAGAA
ATAAGGGAAATAAGAGAAACGCGATATGAAAGCCACTATCCTAATTACTCTTACAGTTTATATTCTCCACCTAACTCTACAATAATTACACAACTGG
GATCGCCACCGACTACTTACTCTCCTACAATTTCATCTTCTCATCAATATGAAAGGGCTCCAAATGGGCAAGCTAATCACCATTATCCATCTTCGAC
TAGTGTTATAACTTTGAAAAATTGCTCTCAATTGAGCTTGATTCAAAATTCTAATATTAATGGGCAGTTGGTACCACAACATAGTAGTCTAAGTCCT
GATGGAACATGCAGTCTAATTGCACCTATGAGCCCTAATTCACAATCGTCTCGTGGATATAGAAGTCTACCTTACCCATTAAAGAAAAAGGACGG
GAAAATGCATTACGAGTGCAACGTATGCTGCAAAACATTTGGACAACTATCTAATTTAAAGGTCCATTTACGAACACATTCTGGGGAAAGACCTT
TCAAATGCAACGTTTGCAATAAATCCTTCACACAACTGGCTCACCTTCAAAAGCATCACTTAGTACATACCGGAGAAAAACCACATCAATGTGAT
ATTTGTAAAAAACGATTTTCATCTACATCTAATTTAAAAACACACCTGCGGTTACATTCAGGACAGAAACCATATGCTTGTGATCTCTGTATGCAA
AAATTCACACAATTTGTCCATTTAAAACTTCACAAGCGACTGCACACAAATGACAGGCCATACGTTTGTCAAGGATGTGACAAAAAATACATCAG
TGCCTCCGGACTACGAACTCACTGGAAAACGACAAGTTGTAAGCCCAACAATATTGAGGAAGTTCTCGCCATCACGAATGCAGCAAACACCGA
ATGCATAGGTAACATCGACAAAGACTGCCAAGAAAGAGAGGGGCACGAAGCATATGAAGTCCACTCTGCTACGCAGGGAGGACTGGTGGGAAC
CGGTGGGGCAAGAGTGTCAATTTCGCACAGTGAAGCAATAATGGGTGGGCAAGCGCACACCACCACACCGCATCTAGCAGGCCATGGGCAGCT
TTCACCAGGCCCGGGTGTTTCACAATATCGTGGATTAATGCCTCCTGCTGATCATGAACAACAACACTACGGTTCAGCTCAGAGCGAGTCCCGGC
CGAGTGTTATAGAATCAAACCAACCGCTCATCATAGAGTGCACTTGA

>*Heliconius_Melpomene* HMEL007072

ATGAGAGAATTGAGTAAATATGAGAATATAGATAAGCGTACTAAAATTAATAAAAACCATTCAACAAATTTAAGCATCAAAATGAGGCTGTCAAC
GAGTGAAGAAATCCCTACTACGACGGTTGGCCGTACAGCAAGTCCCGGCGTGGGCGCTGTTGGTGCTGTAGGAGGCACGGAGTCATATGATGTA
ACGCGCATGCGTGAAGAAGAGTTTGAAAGGCTCACAGTGTACATAGTGCCTGACGTGCCGTGTGAACGCGGCGTTACAAACAGAGCCGAAAGG
ACACTTCCGCGCAGTTTGGCACTAAGACCATCTGCAGTATTATCAACACCTAACACGCCGACCGAAGGCGTATGGAGTACAGGAGTTATTCCACG
AGGCACACGATTTGGACCTTTTGAAGGAACACGGACCCCTAATAAACCAAATGACAAAGTCTCTTGGCGATATTATTGGCGGGTACAAGAAACT
GTCAGACATCATACTTATGGAAATATTAAGATATTCAAAGATAGTGATTATTATTACCTGGATGGGTCAGACACGAGTGTCGCCAACTGGATGCGAT
ACGTGGCCTCAGCATATTCCCTCTCCGTAATGAACCTCGTTGCGTGCCAACACCAAGAACATATTTATTTTTACACAATTCGTGACATAATGCCAA
ACGAGGAATTAATGGTATGGTATTGCAAAGATTTTGCGACCAGGCTCGGCTACGATATTGACCCAGAAAGAGCTACATATTCTATATGTAAAGAAG
AAACTATAAAGAAAGCTTACGGATTACCACCTGCACCAGTACATCCTGAAATTGCTTTTAATCACATGAAATATGTTCTTGGACTTACAAATGCTA
AAGAACTCCTGGAAGCGGAAATCCCATGTAGACGAAGGAAACGCGAAGCGAGTGACAAATCATTGCATGAAAATAACAATCTTCCGGCACGTG
ATGAACTTAGAAGTACAATAAAAAATGAAAGAATAGTGATGAGACCGGACATTCCCTCTAATATTATTCATCAAAAATCTGATCCTATTATCAATGA
CCATCCTTCAATATCTCCTAATACCTTAATGCAACCACCTTCACCAGTAAAAGCACATCACAGGGATAATCAGACACTCATAATTCACCAACACAA
AGAAAGTACGTCACATGTTGTTATACATCAACTTGAAAATCCAAGACTTTATAACAATCGATCACCAGCTCAAAATATCCACATGAGAGATATGCG
AGGACCTTCTCCAATGGGACCAAATGTTCCAGAAAAGCTAGGTAGCAAATCACCTAGCTTTACCCATTGTGTACCTACACATTACGAACACCAGC
TAACACCAAATGATGGGTCAGTAAGATCAGATGAGGGATATCACAGCAATGGCTACCATGATGAATTTACTCCTCCTGAAGATTCAAGCGACTCA
GATGCTGAAAATTATGTATTAGATTGTTCAAATAAAACTAATGAACCAAAAGAAACAGTTATAGTTCAAAAAATTGACCAAGACATGCACAGAAA
TGAATACAGGAAAGTTAAAATTAAAATGCCAAGAGCTTATGCTCTAAATAAAGATATGGACTGTGAAAGTGAAAAGGAGTTAATGATAACTGAAG
AAGTTATACATAGTCCCCCACAAAACGTGTCACCACCACGCCGTGACCCAGCTACATCGACTGTTATTGTTCTTGAGTCATCTCAAAACACTGTA
GTTCCAATAAATAAACCATACTATGAAGATGGGACAGTTTCACCAACTCCACAACCAGCTTTCATGAGATATTCACCACCAGATACTAGAATTCTA
GAAACTATTCTAACAGGCAATAGAATTGACACTAATAATAACAATGACCCAAACAGAAGGCAACCAAATGCTACTCCGCCACCATCATCACCAAC
AGAAATGGCATATTCGTACAAAAAGAGCCAAAGGTATGGTAATGCCTGTAGTCCTGATTCTAGTTCCAATTTAACACCTTTGCCATCTTTACTGCC
ACTACCACAGCAGTTACCAGCACCTAATTCAGTTTCTGTTTATTCTTCATCACCTCCTCACTCGATTCCTCATACTACCGAAATTCGGGAAATTAGA
GAGATTAGAGAAATTAGAGAAACGCGATATGAAAGTCATTACCCTAATTATTCTTATAGTCTCTACTCGCCGCCTAACTCAACAATAATAACGCAG
TTGGGATCTCCACCTAGTACATATTCTCCAGTTTCATCATCACATCAATATGAAAGAAATCAAACTGGACAAAATAATCATCATTTTCCTTCTTCAA
CGAGCGTCATAACGCTTAAAAATTGTTCACAATTAAGTCTTATTCAAAATTCTAATGTGAATGGTCAACTAGTACAACAACACAGTAGCATGAGCC
CAGATGGTACATGCAGTTTAATGGTCGCACCATTGAGTCCAAATTCACAATCGTCGCGAGGTTATAGAAGTTTACCATACCCTTTGAAGAAGAAA
GATGGAAAAATGCACTACGAATGCAACGTTTGCTGTAAAACATTTGGTCAATTATCGAATTTAAAAGTGCACTTAAGGACGCACTCAGGAGAAA
GACCTTTTAAATGCAACGTTTGCAGTAAATCTTTTACCCAGCTGGCTCATTTACAAAAGCATCATCTAGTGCACACGGGTGAGAAGCCACACCAA
TGTGACATTTGTAAGAAAAGATTTTCGTCTACATCGAATTTAAAAACACACTTACGTCTCCACTCGGGCCAAAAGCCGTACGCTTGTGATTTATGC
ATGCAAAAATTCACCCAATTCGTTCATCTAAAACTTCACAAGCGACTGCACACAAATGATAGACCTTACGTCTGTCAAGGATGCGACAAAAAGT
ATATTAGTGCGTCAGGCCTCCGTACACATTGGAAAACCACTAGTTGCAAACCGAATAATATTGAGGAGGTCCTAGCGATCACCAACGCCGCCAAC
ACAGAATGCATAGGTACCGTCGACAAAGACTGCCACGAGAGAGAGGGGCACGAGACATACGAGGTGCACTCGGCGTCGCACGGAGGGCTGGT
GGGAGCCGGTGGGGCGCGCATGGGAATGTCGCACAATGACGCGCTGATGGGAGGGCAGGCGCACACGACCACGCCGCATCTGACCATGGGCC
ACGGGCAGCTCTCTCCCGGTCCGGGGAGCCAGTACCGGTCGTTACTCCCGCCGGGATCCGACCACGAGCAGCAACATTACGGCTCCGGTGGTG
GAGAGACCAGGCCGAGCGTTATAGAATCGAGCCAACCTCTTATAATCGAGTGCACCTGA

>*Tribolium_castaneum* TC014741 XM_008196205.2

ATGAGAGAGGACATGATGGATGAGGTAGCTGTAACGTCCGCCAGCGAATTCGACTTGGCCAATATCCATGAGGAAGAGTTCGAATTGCATACCA
CCTACATCGTTCCGGATCTTCCGGTTGAGCCCGGAACGCCGAACCGGGCGGAAGCTACCCTTCCCCGCAACCTAGTGCTGAAGACATCGCAGGC
AATGTCTGATGCTCAGGGCGTTTGGAGTACGGGGTACATCCCACGAGGGACGCGCTTTGGGCCTCTCGTGGGAGAAATCTATGCCAAAGATGCG
GTACCAAGCTCGGCGAACCGGAAGTACTTTTGGAGGGTTCAGATGAGAGAGGGCTTTTATAGAAACAGTTCTAACGACAGCAAGATATACAAAG
ATAACGAATTATATTATTATATTGATGGATTTGATACATCTAAAGCTAACTGGATGCGATACGTGAATCCTGCTTATTCCAGCGAATCGCAAAATTTA
ATCGCCTGTCAATACAAAATGAATATCTATTTTTACACTATCAAACCAATTCTTCCCAATCAGGAACTTCTTGTCTGGTACTGCAAAGAATTCGCCG
AAAGACTTAATTATCCCTTGACTGGTGAACAAATGCTACAAAGAATTAGACAACAAGTACAGCAATCTTCTGAAGTGATAACTGTTAGTACGACT
GAACCTCCTGCAAAAGACGGTCCCTACGAACACCAACTAACACCAACTGATGGTAGTGTGAGATCAGACGAAGGATATCATAGTAATGGCTATC
ACGACGATGCTTTCACACCACCTGAAGATTCGAGTGATTCAGATAGTGAGAATAATTACGTTCTTGATTTTAGTAAAAAACCAAATTCGAAAGAA
ACCGCGGTAATAAAACCGGTTTCTCCCGAAACCCAAAAGAACGAATACAGGAAAGTGAAAATAAAAATCTCGAAAGCTTACCACTACAAGTCG
AAAGGAGTGGAAAGCGACCACGAAATGGACAAGGAATCCCACACTGACGCTGCAGTACCTGAAGTAACAACACTAACCCCGAGAATAATCACG
CCCCCAAGTACAGTAATTGTGATTGATTCTCCTCCACCTGAAGTTCCTAACAATAAACCTTTCTACGAACCTGAAGTCAAGCCCCAGAATGTTATT
TCGCGGTATACGCCGCCCACTTCTAGCATCCTTGAGAATATTTTGCTAAGGAATCGAATTGATACCAATAATAACGACAACAATCAGAGACAACCA
AACGCAACCCCTCCTCCAAGCTCCCCCACCGAAATGGCTTATTCATACAAGAAAAGTCACCGGTATGGCACTGTCCCCTGCAGTCCAGACTCAA
GTTCAAATGTCCAAGTTCAAAACATGCTCCCTCCTAGTCCCGCAATGCTTAAGAACCACCCCCCAAGTCCCGTCCAGCCCTTGTACGTCCCCCAC
GACAACTACCCTAACCACATGCAACCCTCCAACTACTACAACATTTACCCTTCAAACGGCATCCCCCATAGTACTATCACCTCCACCCCCACAACC
ACCTACTCGCCCCCAGCCACCCACCACCACAACGGTTCGAATCTAATGATCCCAGCGTCCCACATCAACCTCACCCACCACCTCCTCACACCCCT

ACCCCCACTGCAAACCAGCGGCAGGTCCTCCCCCTGCTCGCGGAGCCCCGAGGGCGGGTCCCCCGGTAGCCCCAACACCAACCAGAACCGCG
GCTATCGCAGCCTGCCCTACCCCCTGAAGAAGAAGGACGGGAAAATGCACTACGAGTGCAACGTTTGTTGCAAAACCTTCGGCCAGTTGTCCA
ACTTGAAGGTCCATTTAAGGACACACAGTGGCGAACGCCCCTTCAAGTGCAACGTCTGCACCAAGTCCTTCACGCAGTTGGCCCACCTCCAGA
AGCACCACCTGGTGCACACTGGAGAAAGGCCACACGAGTGTGGCATTTGTAAGAAGCGATTCTCCTCCACGTCCAACCTTAAAACCCACTTGA
GGCTACATAGTGGCCAGAAACCCTACGCGTGCGATTTCTGCCCCGCCAAGTTCACGCAGTTCGTCCACTTGAAGCTCCACAAGAGGCTGCATAC
GAACGAACGGCCGTATATTTGTCAGGAGTGCGGGAAAAACTACATCAGTGCGTCTGGCTTACGGACGCATTGGAAGACGACGAGTTGTAGGCCG
AACAATATTGATGAGGAGTTGAACGGCGAAGGCTCGCCTAATGGGTACTACGAGTACGCTGGGTCGGATGGAAGTCTAGGATCGATAGATATGAA
CAAGGACATGCATGAGGATTCGAAGGAGTTTCTGGAGGTGCAGCAGGCGCCGATAGGGCACGCGGTGCTGCACCCCGGGCTGTCGAGGCCTCT
GCCGCCGCAGCAGCGGCAGCCCCCGCAGCCCCCCATCCACCCCGGGAAGGAGACGCGGCCGAGCGTGATCGAGTCGTCCCAACCCCACATAAT
TGAATGTACCTAA

>*Drosophila_melanogaster* CG5249 NM_001274514.1

ATGCATGGACATCACAACCACAACCATCTGCCGCAGCAGCAGCCACAAAATCCGCAGGAGCAGCAGTCGGGTGCCATCATGAAGGCAGCCAAC
CAGCCCCACCATCCCGCCCACAGCCATCCGCTCAATCACCCACATCCGCACCAGCATGCCCAGCCACATCCGCATCCACATCCCCATCCGCATCA
CAAGCTGTCGGCGAGAAGGGCGCGCAGTCGCAGTCGAAGTCGCAGTCCGACGCCGGATGGCAATCAGGACTACGATGTGACCAGGATGCGGG
AGGAGGACTTCGAGCGGCTGGCCGTCTACCTGGTGCCGGATGTCCAGGCGGAGCGGGGACTGCCCAATCGGGCGGACAAGACGCTGCCGCGC
AGCCTGACGCTCAAGTCATCCATGGTCTACTCAACGCCAAATGTTAAGACTGAAGGAGTTTGGAGCAGCGGGGTCATCCCACGTGGCACTCGCT
TTGGCCCATTCGAAGGCATTCCAACCTCTAATTATCCCAATGATAAGAATAAGGCGCGCTATTTCTGGCGGGTGCAGGGAACACGCCACATAACC
TACGGGAATATTAAGATCTTCAAGGACGATGACTACTATTACTTGGATGGTTCCGATCGCTCGCAGTCCAATTGGATGCGCTATGTGGCCTCCGCT
TATAGTTTATCCGTAATGAACCTGGTGGCGTGCCAGCACCAGGAGAACATCTATTTCTACACCACCCGCGACATTCTGCCCAACGAGGAGCTAAT
GGTTTGGTACTGCAAGGACTTCGCCAGTCGCCTGGGCTACGATGTGGATCCTGAGACGACGATCTTCGGCGCATGCAAGCAGGCTGTGGAAGCG
GAGGAGGAGGCGGACGAGGAGGAGGAAGAGGCGGAGGGAGAGGACGGAAAGCCGCGATACTCGATGCCAGCTCCGGAAATTCCACCCGATG
TGGCCGTGAGTCACATAACCTACGTCATGGGTCTCCATCTGCCTGTGGGAGCGGGAAATGGTCCGGCGAATGGTTCGGTTGCGGGTTCCGTTTCC
GGTGCCACACCTCCACCACCGACTGCCACGCCATGCTGCCGCCGCTCCAGTCCACCCGCCCACGTCAGCACCACCACCTCCACTTGCAACGCCC
ACCACCCGCACATCCAGCATGGTCGACATGCCTCCGTAATCATCGGCCAGGATCGATCGCCTATGGCCAGCAGTGACAAGGATACGGCGGGATCT
CCCCTTTCCGGTCTGGACCATCAGATGACCCCGCAGGATGGCAGCGTGAGGTCTGTGAGATCGGACGAGGGCTACCACAGCAACGAGTGCCAC
GAGGATGGACTCACACCACCCGAGGATTCCAGCGACAGCGAGAGTGAACATAACTACGTGCTGGACTGCTCGAAGAAGGCAATTGCTCCCAAA
GAGACAGTGATTGCCCAGGCCCAAAAGCCCAGCAGTTCTCCACCGGCAGTGGTAATGGCCAACACGACTACCAACTCGATGATTAGCCACAGTT
CACCCACTGCGACGCCCATTTGCGAAACGGACAAGAATGAGTATAGAAAGTTCAAGGTGAAAATGCCGCTGAAGTACGAGTTCAAGAACAAGA
CCTGCGTAAAACAGGAGCCAAGCCTTAAGGAAGTGGACCAGGAGATGTCCTTGCCGCAGGAGGAGGAGGAGGATCAGGTGATGCATCCGGAA
CCCGATTCAATTTGCCCATCGACCACCACCCATCTGGGCGATGAGCACATGCTGATGATGGAGCGGGAACGGGAAAGGGAGCGCGAAAGGATC
CAGGAGAGGGAACCATCCAACCAGCAGCCCGCCTCCTCCACGGTCATCGTACTGGAACACAACTCTGGCGGTCAAGCCCGCACCATTGTCCCA
CTGAGTAAACCCTACTACGAACCAGATCCTCCCGGCGAACGCTATATGCGATTTGGCCAGCCCTCTTCCAGCATCCTGGAGACTATCCTCACCAG
CCAACATCGCTTGGAGGCGGCAGCTGCTGCGGCAAATGCCTGCCGACAGGCCAATGCCGCCACACCACCGCCCACATCGCCCACGGAGATGGC
CTACAGCTACAAGAAATCCCAGCGGTACGGTAATGCCGTGAGTCCGGATAGTAGTTCCAATCTGGGACAGAATCCCGAGCAGCTTTCCTCGTCG
GCAGTGGTGGTCGGCGAACAGGAAATGACCAGAGCCACCATGATCAAGGGAGAGTGCTCACCACCACCGCCTAGCCACCATCATCACAATGTC
ATCTTCTCGCCCTCGAGACATGCTGCTTATCTGGGAGCCGGCGAAGCGGGTGGCCACTCACCGAGTCCGGGTTATCCTGGCTATCCCCACTATGG
AGCTGCAGCCACCTCGACCTTCCACTCACCACCACATAGTTCCCATTCCCCGTTCGATCGCCAGTCGAATGCATCCAGTGGTGCGGGTTCGGCCA
CAAATCTGCATTTATTGCAAACCAGCACCCAAATGCTGAACCATCCGCTGATGCAGCCCCTCACACCCCTGCAGCGTTTGTCCCCGCTGAGGATA
TCCCCACCCAGCAGCCTCAGTCCTGATGGCAACTCGTGTCCCCGCAGCGGCAGCCCCTTGAGTCCCAATTCCTTGGCCTCCAGAGGCTACCGAT
CGTTGCCTTATCCGCTCAAGAAGAAGGATGGCAAGATGCACTACGAGTGCAATGTGTGCTGCAAGACTTTTGGCCAATTGAGCAACCTCAAGGT
GCACCTGAGAACCCATTCCGGCGAAAGGCCCTTCAAGTGCAATGTGTGCACGAAGAGCTTCACCCAGTTGGCGCACCTCCAGAAGCATCATCT
GGTGCATACGGGCGAGAAACCGCATCAGTGCGACATCTGCAAGAAGCGTTTCTCGAGCACCTCCAACCTGAAGACCCATTTGAGGCTGCACAG
TGGTCAGAAACCATATGCCTGTGATCTCTGCCCGCAGAAGTTCACACAGTTTGTGCATCTGAAGTTGCACAAGAGACTGCACACGAACGACAGG
CCCTATGTGTGCCAGGGTTGCGACAAGAAGTACATCAGTGCATCCGGCTTGAGGACCCACTGGAAGACCACCAGCTGCAAGCCCAACAATCTG
GAGGAGGAGCTGGCCATGGCAGCAGCGGCAACATCGGAGTGTTTGGATAAAGACCATCCCGAACCGGACTCCCGAGAAGCCTACGAGCAACTG
ACGCAGCACATGCATCCTGCTGTGCATCCGGGATTGAGGCACCTCTCGAGTGGGGGTCAATCGCCACCGCGCCTGATACCCCTGGGCAATCACAT
GGCGCCACAACAGCAGTCGCATCAGCAGCAGCAACAGCAGCATCAGCAGCAGGGAGTGCCACCGCCCCACTTACTGATGACCCAGCATTCGGT
GGGTCCGGCACCCATGCTGCTGACCACCGCCAGCCAGTTGCCACCACCGCCGCCACATCATCAGCAGAACTCGCCCAGCCGTCTGCTGCAACAT
GGCCACGCACATCCGTTGCAGATGCAACAGCAACAGCAGCAGCAACAGCAGCACTCGCCCAAGGGACTCAAATCCCTGCCGGAATCGGGGGT
GTATCTGCATGGACAGCATGTGCAGGCCCAGGAGAGCAGACCATCGGTCATTGAGTCCTCCAACCAGCCCATGATCATCGAATGCACGTAG

> *Papilio xuthus* XM_013322813_1

ATGAGACTGTCAACAAGCGAAGAAATTCCGAGCACGACTGTGGTCGGCCGCACCGGTAGCCCTGGCGGTGGAGGTGCTGCATCGGCCGCAGCA
GTGGTCGGGACCGGAGCTTACGATGTGACGAGAATGAGGGAGGAGGAGTTTGAACGGCTCACTGTATACATAGTGCCCGACGTGCCGTGTGAG
AGAGGGACACACAACAGAGCAGAGAAAACTTTACCCAGAAGCCTCGCACTAAGACCTTCCGTAGTGCTGTCGACACCTAACACACCGACCGA

AGGAGTATGGAGTACCGGCGTTATTCCCCGTGGCACTCGCTTCGGCCCGTTCGAAGGCACCCGAACGCCAAACAAACCTAACGACAAAGTTTCC
TGGCGATACTATTGGAGGGTACAGGAAACTGTCAGACATCATACTTATGGAAATATTAAGATATTTAAGGATAGTGATTATTATTACCTGGATGGAT
CTGATACGAGTGTTGCGAACTGGATGCGTTACGTGGCCTCTGCCTACTCCCTCTCCGTGATGAACCTCGTGGCTTGCCAGCATCAAGAGCATATAT
ACTTCTACACGATCAGAGATATTATGCCAAATGAAGAATTAATGGTTTGGTACTGCAAAGACTTTGCGACGAGGCTAGGATATGACATCGATCCCG
AAAGAGCAACCTACTCCATCTGCAAAGAAGAAACTATCAAAAAAGCATATGGCATACCCCCTGCCCCAGTTCATCCCGAGATTGCATTTAACCAC
ATGAAGTACGTGCTCGGCTTAACGAACCCTAAAGAACTCCTAGAAGCTGATATTCCCACCCGAAGAATAAAACGTGAATCTATTGAAAAGGCAC
ACATTGAAAATTATACACCACGTGAAGATCCTCGAATTACCAATAAGAACGAAAGGACAATTATAAGAACCGATGTATCTAGTAATATTATGCATC
ACAAATCAACAAATGATATTCAAATGGCCAGTGAACATAGAACGATATCACCGAATGGTCTCATGCAGCCTCCATCACCAGTTAAAATGCACCAC
AGAGATAATCAGACTTTAATAATTCATCAACACAAAGAGAGTACATCGCATTTAGTAATACATCAACTTGATAATCCCAGATTACATGCCAACAGG
TCACCAGGACAAAACATACATATGAGGGATATGCGAGGACCTTCCCCAATGTTACAACATCACCAGGACAAACTAGGAAGCAAGTCGCCGGGCC
TAACGCACTGCAGCGCTCAACACTATGAACATCAACTAACACCCAATGACGGATCGGTAAGATCAGATGAAGGTTACCACAGTAATGGATATCAT
GACGAATTTACACCACCAGAGGATTCCAGCGATTCAGACGTAGATTCCAACTATGTTCTTGATTGCTCGAATAAAACAAATGAACCAAAAGAAA
CTGTCATAGTGCAAAAAATGGACCAAGATATGCACAGAAATGAATACAGAAAGGTTAAAATAAAAATGCCAAGGGCATACCAATACCAGCATCA
CAAGGACATGGACTGCGACAGTGAAAAAGAACTGATAATTGCCGAAGATGTAGCTAACATATCACCACAGAACTTATCTCCGCCCAGACGTGAC
CCGGCTACTTCCACTGTTATAGTCTTGGAGTCATCACAAAACACAGTTGTTCCTATTAACAAGCCTTATTATGAAGATGGTACAATATCTCCAACAC
CACAACCGGCATTCATGAGATACTCTCCGCCTGACACAAGGATATTAGAAACCATATTAACTGGTAGTAGAATCGACACAAATAACAACGATCCT
AATCGCCGTCAACCTAACGCGACGCCACCACCATCGTCTCCTACTGAAATGGCATATTCTTACAAAAAGAGTCAAAGGTACGGGAACGCTTGCA
GTCCTGATTCTAGTTCTAATTTAACACCCTTGCCATCACTACTGCCTTTACCACAACAACTACCGGCTCCCAACGCTACGTCAGTCTATTCATCGTC
GCCGCCCCATGCCATCCCACATACGACAGAAATACGGGAAATACGAGAAATACGCGAGATAAGAGAGACTAGATATGAGAGTCACTACCCAAAT
TACTCATACAATTTATACTCTCCACCAAACTCTACTATTATAACGCAACTTGGATCACCACCTAGCACTTATTCCCCAACCGTGTCTTCCACTCACC
AGTACGAAAGGTCACAAAGCGCTCAAAGCAATCACCACTATCCCTCGTCGACCAGCGTTATAACACTTAAAAACTGTTCACAATTGAGTTTAATA
CAAAACTCAAATGTCAATGGTCAACTGGTACCACAACACGGCAGCATGAGTCCGGATGGTACTTGCAGTCTCATGGGCGCCCCAATGAGTCCAA
ACTCTCAGTCCTCTCGCGGTTACAGAAGCCTGCCTTATCCATTAAAGAAGAAAGATGGAAAAATGCACTACGAATGTAACGTTTGCTGTAAAACG
TTCGGCCAACTCTCGAATTTGAAAGTGCATTTGCGGACACACTCCGGGGAACGGCCGTTCAAATGCAACGTGTGCAATAAGTCCTTCACGCAGC
TTGCGCACTTACAGAAACATCATCTCGTTCACACCGGAGAAAAACCACACCAGTGTGATATCTGCAAGAAGAGGTTTTCATCCACTTCAAATTTG
AAGACACATTTAAGACTGCACTCAGGTCAAAAACCTTATGCCTGTGATCTGTGTATGCAAAAGTTCACTCAATTTGTCCATCTGAAACTTCACAA
GCGGCTGCATACGAACGACAGACCATACGTGTGCCAAGGGTGCGACAAAAAATATATAAGCGCCTCTGGACTAAGAACACATTGGAAAACCAC
AAGCTGTAAACCCAATAATATCGAAGAAGTTTTAGCAATCACCAACGCTGCGAACACTGAATGCATGGGTTCCATCGACAAAGACTGCCACGAT
AGAGAGGGGCACGAGGCGTACGAGGTGCATTCTGCGTCGCAAGCGGGTCTCGCAGGAGTGGGCGGCGCTCGGCTGCTGGCCGGCGAGGGCGC
GCTTGGTGGACACGCTCATTCCACCACGCCACACCTCGCGCCGGGCCCACTGTCGCCGCCACACTCGCAGCACGCTCAGCACGCGCAACACGC
GCAGCCCTACCGCAAGCTGCATGGGCAGGCGCAGGAGCACGAGCCGGCATCGCAGCACTACTCCTCGGCGGGTGAGACGCGTCCCAGCGTAAT
AGAATCGAGCCAGCCTCTCATTATTGAGTGCACCTGA

> *Anopheles_gambiae* XM_316619_4

AATGTCTGGAGCGAATCCCCCTCGAAGCGTTACGCCAAACGCGCAGCAGGACTACGATGTTACGCGTATGCGCGAGGAAGACTTCGAGCGGCT
GGCGGTGTACATCGTGCCGGACATGCCCTGCGAGAAGGGTGTGCCCGGCCGGGCGGAGAAAACGCTCCCCCGCAGCCTGACGCTCAAGCCCTC
CCTCGTCCTGTCGACGCCCACCTCCACGACTGAGGGCGTATGGAGTACGGGTGTCATCCCGCGCGGCACCCGGTTCGGCCCGTTCGAGGGCGTC
CGCACACCGAGCCACCCGAGCGATAAGCAGCTGTGGCGGTACTTCTGGAGGATATTCAAAGACAGTGATTTCTACTATCTCGACGGGTCGGATAC
GGCCCAATCGAACTGGATGCGGTACGTCGCGTCCGCGTACAGCTTCTCCGTGATGAATCTGGTCGCCTGCCAGCACCAGGAGCACATCTACTTCT
ACACGATACGCGACATCATGCCGAACGAGGAGCTGATGGTGTGGTACTGTCGCGACTTTGCCAAGCGGCTCGGCTACGACATCGATCCCGAGCG
GGCGACCTACTCGATCTGCCGCGAGGAGGTCGTGAAGAAGACGTACAAGCAGCCCGTCCCGCCGGAGATCGCCTACAACCACGTGAAGCACGT
GATGGGCCTCTACCTGCCGGCGATGGTGCGCAGTACGCCAACGCCCAGTCCCTCACCACCGGCGCCCGCAACCGTCGGTGGTCATGTTCCTTCC
GCGAAGCATCACGCCCTGCACCAGACGGTACTGCGCGAGCCTCGCAATAGCAGCTCGGCGGCGGCTGTCACCGGCGATGAGTCCCTGCGGGAC
CGGTCGCCCATCAGGATAGTGATCCGCGAGCAGAGCCCACTGTTGCGCGAAAGCGTCAAGGAGGTCGATGCCGACTCTCCGATCTCACAGACG
AAAGAGACGGCGTACGAGCATCAGCTGACGCCGAACGATGGGTCGGTACGGTCGGACGAGGGCTACCACAGCCACGGGTACCACGAGGATGG
CTTCACGCCCCCGGAGGACTCGAGCGACAGCGAGAGCGAGCACAACTACGTGCTCGACTGCTCGAAGAAAGCGATCGAGCCGAAGGAGACGA
GCGTCGGCCAGCACAAGGTGAGCACGAGCGAACCGTGCGACAAGAACGAGTACCGCAAGGTGAAGATGAAGATGCCGCTCAAGTACGAGTTT
AAGAACAACAGCAAAAACTTCAAGGTAGAGCCGGAGCTGGAGCCAACCGAGCGCGAGGTGGAGCCGGCGGTGCCCGCGAGCAGCGAACCGG
TGGTGGTGGGGGCGAGCATGCGGGAAGTAACGCCCGCCACCAGCACCGTGATCGTGCTCGACACGACACCGGAGTCGTCGGTGGTAGTGCCGC
TCACCAAGAGCTACTACGAAGCCGAACCGACCTCGACCTCGCCCGCGACCTTCATCCGCTACACGCCGCCCAGCTCGAGCTCGATCCTGGAAAC
CATCCTGACCGGCAGCCAGCGAGCGTCCCCGTCGGTGGACGATCCGCACCGGCGGCAGCCGAACGCGACCCCGCCCCCGACCTCCCCGACCGA
GATGGCCTACTCGTACAAGAAGTCGCAGCGGTACGGGACGGCCTGCAGCCCCGACTCGAGCTCGCAGAACCCGCAGGCCCCCGAACCGGCCGA
ACCGGGCCACGGGCCGAACCGATCTCAGCCGCACCATCACGGCGCCGCCGCGGCGGCCGCCACCTCTCCCTCGCCCTCGCCCATCGTCAGCAG
CGGCAGCCTCTACTCCACCTCGAACGGGTCGGCCAGTGTGATCAACAGCGCTTCCAGCTCCACCTACAGCCCGCCGCTGAACGGTGGCCACTAC
GAGAGACTGACGACCTCGTCGACCACGCCAACCATCCGCTCGGTGTCGCCGCCGCCGCCGCCACCAACGCAACCAGCGCCGGCCCCGGGCTAC
GTGCAGCTCCAGTCGAAGGGGCAGCTACCGCTGGGCCACCCGCTCATGCAGCCGCTAACGCCACTCACGCACATGTCCCCGGGGCGTATCTCGC
CCCCGAGCAGCCTCAGCCCGGACGGTGGTTCGTACTCGCGCAGCGGCAGTCCCATGTCCCCGGGCAGCCCGAACTCGCGCGGCTACCGCAGCC

TGCCGTACCCGCTGCGCAAGCGCGACGGCAAGATGCACTACGAGTGTAACGTGTGCAGCAAAACGTTCGGCCAGCTGTCCAACCTGAAGGTGC
ACCTGCGGACGCACTCGGGCGAGCGGCCGTTCAAGTGCAACGTCTGCACGAAATCCTTCACCCAGCTCGCCCATCTGCAGAAGCACCATCTGG
TGCATACGGGCGAAAAGCCGCACCAGTGCGACATCTGCAAGAAGCGGTTCAGCTCCACCTCGAACCTGAAGACGCATCTGCGGCTGCACAGCG
GCCAGAAGCCGTACGCGTGCGACCTGTGCCCGCAGAAGTTCACCCAGTTCGTGCACCTGAAGCTGCACAAGCGGCTGCACACGAACGACCGG
CCGTACGTGTGCCAGGGCTGTGATAAGAAGTACATCAGTGCGTCCGGGCTGCGCACGCACTGGAAGACGACCAGCTGCAAGCCGAACAATCTC
GAGGAGGAGCTAGCGATGGCGGCGGCCGCCACCTCGGAGTGTTTGGATAAGGACGGCCAGCCCGACGGGGAAGGTGGCCGCGAGTTCTACGA
AATGTCTCACCACGGTTCGCCGGGCGGCGTTGTCGTACAGCAGACGGCGGCCGCACTGTCACCAGCGTCGCACCACCAGCATCACCAGCACGC
CGGCCATCACCACGGTACCGAGCAGGTCATACACAAGGTGTCGGCCGGGAAGATCCAGCAGCAGCAGCACCAGGGACCGTCCCCGCCGAACA
GCAGCAACAACAACAGCCCCAACAGTACCAACCACAATCTAGCACCGTCGCCGCACCTACACTACGGTGGCCATTTGGTGCAGTCGGGTGGCG
GTGGGAATGGTACGGCGAACGGTGGGCCCGGTCCGGCCCAAAGCCAGCACGAACCGGCCAACCGGCCGAGCGTGATCGAGTCAAGCCAGCCG
ATGGTGATCGAGTGCACGTAG

>*Aedes_aegypti* XM_021842088_1

ATGGCCGGAACGCAACCACCCCGGAGCGTGACGCCGACTGTCCAGCAGGACTACGACGTGACGCGTATGCGCGAGGAGGACTTTGAGCGGCT
GGCCGTCTACATCGTGCCGGATGTGCCCTGCGAGAAAGGCATCCCCAGCCGGGCGGACAAAACCCTGCCCCGCAGCCTCACGCTCAAACCCTC
GCTGGTCCTGTCGACGCCAACCGCGACGACGGAAGGAGTATGGAGCACCGGAGTGATACCCCGCGGTACTCGATTTGGGCCCTTCGAGGGCGT
ACGGACACCCAGCCATCCCAGCGACAAGCAGTTGTGGCGGTATTTCTGGAGGGTCCAAGAGCTGCGACACGTTTCCTATGGGAATATCAAGATA
TTCAAAGACAGCGACTTCTACTACCTGGATGGCTCGGATCCAGCGCAGTCCAACTGGATGCGGTACGTCGCATCGGCGTACAGCTTCTCGGTAAT
GAACCTGGTGGCCTGTCAGCACCAGGAGCACATCTACTTCTACACAATCCGGGACATCATGCCCAACGAAGAACTGATGGTTTGGTACTGCCGG
GACTTTGCCAAACGGCTGGGCTACGATATTGATCCCGAGCGGGCGACCTACTCCATCTGTCGGGAGGAGACGCTCAAGAAGACGTACGGTGCAA
TCGCTAAGGCGCCAGTACCGGCCGAAGTGGCCTACAATCACGTCAAACACGTTATGGGCCTTGGGATGTACATGCCGGCGATACGGAACGCCCC
AACGCCTAGCCCTTCGCCACCTGCCGCAGTACCCAGCACCACAACAGTTGTAGTTCCTTCCAAGCACCAGATGCATCAGACGGTGCTGCGAGAG
CCGAGGACAGCGGTAGAACACCACCGGGACCGATCGCCGGTCAGGCTTATCATCCGGGAACAAAGTCCACTGAGGGAAAGTGCAAAAGAGGT
CGATGCCAACTCACCGATCTCACACAACGGCAAAGAGGCTCACTATGAGCACCAATTGACGCCGAACGATGGTTCGGTAAGGTCTGACGAAGG
ATACCACAGCAATGGCTACCACGAGGATGCTTTCACCCCTCCGGAAGACTCCAGTGACAGTGAGAGTGAGCATAACTACGTGCTGGATTGCTCG
AAGAAGGCGATCGAACCTAAGGAGACCAGCGTTAGTCAGAAGGCTGAGATTTGTGATAAAAATGAGTACCGGAAGGTCAAAATGAAAATGCCA
TTGAAGTATGAGTTCAAGAACAATAAAAACTTCAAATCACCTAGCGATAAGGAGGACATAGTGGAGGAAGAGGAAGTTGTCACGACGAGGGAG
GTTACTCCGGCCACAAGCACAGTTATTGTGGTAGACACCACTCCAGAAACGACGATTGTCCCCCTAACCAAAACATATTACGAAGCGGAGACTT
CCACATCTCCAGCAACTTACATCCGATACTCTCCGCCAAGTTCAAGTATTCTTGAAACAATCCTCACCGGCAGTCGACCACAGGTGGATGATCCT
CACCGAAGGCAACCGAACGCTACGCCGCCTCCAACCTCCCCAACAGAAATGGCCTATTCGTACAAAAAATCACATCGGTATGGTACCGCATGCA
GTCCAGACTCGAGTTCACAGAATGCCGAACGCGAGATCAGGGAAAGCGAACCACAAATACTACAGGACACCAAGATCATCTCCATAGATCATCG
TGAATCTCAGTCACCGACTTTGATCTACAGCAGCGAACATCGGCCGCAGTCGTCGCACTCGCCTGGTCCCTACGAGCCCTCCCCGCATAATTACG
CCATCTATCCCTCACCGAACGGAACAGTGATCAACAATGCTTCCAGTTCGACCTACTCGCCACCTTTGAACGGTGGCCACTACGAGAGACTGAC
AACGCACACAATCCAGTCCCCTCACGGATTCGTGCAACTCCAAGCCAAGGGACAACTTCCTCTGGGACATCCGCTGATGCAGCCACTTCCCCCG
TTGACGCACATGTCTCCCGGAAGGTGTTCACCGCCCAGCAGCCTCAGCCCGGATGGTGGTTCCTACTCGCGAAGTGGCAGCCCAATGAGTCCGG
GAAGTCCAGGATCCCGAGGCTATCGAAGTCTGCCATACCCACTTCGCAAACGAGACGGCAAAATGCACTACGAATGCAACGTCTGCAGCAAGA
CCTTCGGGCAGCTGTCCAACCTTAAAGTGCACCTGCGAACTCACTCAGGCGAGAGGCCCTTCAAGTGTAACGTCTGTACCAAATCCTTCACCCA
GCTTGCCCACCTACAGAAGCACCACTTGGTGCACACCGGCGAGAAGCCGCACCAATGTGATATCTGCAAGAAGCGGTTCTCCTCCACGTCGAAC
CTGAAGACCCATCTGAGGCTACACAGCGGCCAGAAACCCTACGCATGCGATCTGTGCCCGCAGAAGTTCACCCAGTTTGTGCACCTGAAGCTGC
ACAAGCGACTCCACACCAACGATCGGCCGTACGTATGCCAAGGTTGTGATAAGAAGTACATCAGTGCTTCGGGACTGCGAACCCACTGGAAGA
CGACGAGCTGCAAGCCAAATAACCTGGAGGAGGAGCTGGCGCTGGCCGCTGCTGCCACTTCCGAATGTATAGACAAAGATCAACCGGACAGCA
ATGATCACCGAGAGTACTACGAACTGCAGAACCATCCGCCCTCTCCAGTAGCTCCTCATCAACTGAGGCACGTTGTAGTAGGTCACGGTTCTCCC
CCAACTCTCCTCGGCATGTCTCCGCAACATCCAGGCCATCTGATCCAGGTTCACCCGGGATCGGACGCGATCATTCAAAAAGTAGGCAAGCACC
CCTCCAGTGGCAACAGCCCTTCCTCCACTACCACCACGAACAACAACAACCCAACCATGGTGAATGGCAACGTGCATTACTTGGGAGGCCCCCT
GCCACCTCAGGGACCTCTGCCACATCTACAGCATCACCCCCATCACCATCCCGCGTCCCATCAACATCAACCGCCTCAGATCAGCGTTATGGGGC
ACCATGGAGGCGGGCCGGCTCCGCCCAATTCGACGGAACCGCCAACCAGCCGACCCAGCGTGATCGAGTCCAGCCAGCCAATGATCATCGAGT
GCACGTAG

>*Culex_quinquefasciatus* XM_001848998_1

ATGGCCGGAACGCAACCACCCCGCAGCGTGACGCCGAACGTCCAGCAGGACTACGACGTGACGCGGATGCGCGAGGAGGACTTTGAGCGGCT
GGCCGTGTACATCGTGCCGGATGTGCCCTGCGAGAAGGGCGTCCCGGGCCGGGCCGACAAAACGCTGCCCCGGAGCCTCACGCTGAAGCCCTC
GCTGGTGCTGTCGACGCCGACGGCGACGACGGAAGGAGTATGGAGCACGGGCGTGATACCGCGCGGGACGCGCTTCGGCCCGTTCGAGGGCG
TCCGGACACCGAGCCACCCCAGTGACAAGCAGCTGTGGCGGTACTTTTGGAGGATATTCAAAGACAGCGACTTTTACTACCTGGACGGCTCGGA
CCCGGCCCACTCCAACTGGATGCGGTACGTGGCGTCCGCGTACAGCTTCTCCGTGATGAACCTGGTCGCGTGCCAGCACCAGGAGCACATCTAC
TTCTACACCATCCGGGACATCATGCCGAACGAGGAGCTGATGGTGTGGTACTGCCGGGACTTTGCCAAGCGGCTCGGCTACGACATCGACCCCG
AGCGGGCCACCTACTCGATCTGCCGCGAGGAAACGCTCAAGAAGACGTACGGAGCGATCGTGAAGACGCCGGTGCCGGCGGAGGTCGCGTAC

AACCACGTGAAGCACGTGATGGGGTTGTACCTGCCGGCGGCGACGGCGACTGCGGTGACGGCGTCCGCGATTCGGAACACACCGACGCCGAGT
CCGTCGCCACCGGTGACGACGACGACGGCGACGGTGGTGGCGAGTGCCACCACGACCGTCGTGGTTCCCTCCAAGCACCTGCATCAACACCAT
CACCATCATCTTCACCAGCAACAGCAGCAGCAGCAGCAGCAACACCACATTCAGGTGCACCAGACGGTTCTGCGGGAGCCGAGGAGGGTGAA
GGAGGAGCTGCGCGATAGGTCACCGATCCGGATCGTCATCCGGGAGGAGCAGAGCCCATTGCGGGAAAGTGCAAAAGAAGTCGTGGACGCGG
ACTCGCCAATTTCTCAAACCAGCAAGGACAACGGTCACTACGAGCACCAGCTGACGCCGAATGACGGGTCGGTGCGGTCGGTTCGGTCGGACG
AGGGCTACCACAGCAATGGGTACCACGAGGACGGACTTACGCCACCGGAGGACTCCAGCGATAGCGAATCCGAGCACAACTACGTGCTGGACT
GCTCGAAGAAGGCGATCGAGCCGAAGGAAACGAGCGTCACCAGCCAGAAGGTGGACAGCTGTGATAAGAATGAGTACCGGAAGGTCAAGATG
AAGATGCCGCTAAAATACGAGTTCAAGAACAACAAAAACTTCAAGCAGGAAGAGCTCGTCGAGGAGGAGGTCGAGGTTGTGACCACCCCGGC
AACCAGTACCGTGATCGTGATGGACACCACCCCGGAAACCACGATCGTCCCAATCACAAAGACGTACTACGAAGCGGAAACGTCCACCTCCCC
AGCGTCAACCACCTTCATCCGGTACACACCCCCCAGCAGCTCCAGCATCCTCGAGACGATACTCACCGGCGCACGGCCCGTCCCGGACGATCCT
CACCGGAGGCAACCGAACGCCACCCCGCCTCCAACCTCCCCCACGGAGATGGCCTACTCGTACAAAAAATCCCAGCGATACGGAACCGCCTGC
AGTCCGGACTCGAGCTCGCAAAACCCCGTCGAACCGAGCGACCCCCCAACCCTCACCACCACCCTCCAAGTGATCCAGGAGTCCAAACTCATC
GCCGAACACCGCGAATCTCCCCCAACCATCATGTACGACTCCCCGTACGAACCCTCCCCCCAGCACCACCACCCGTACTCCATGTACGCCACCTC
CCCCAACGGAACCGTCATCAACAACGCACCCAGCTCAACCTACTCGCCACCCCTCAACGGTGGCCACTACGAGCGACTAACCTCATCCATCCCA
ACCACTGCAGCCGGATTCGTCCACCTCCAAGGCTCCAAAGCCCCCGGACTCCCCCTCGGCCACCCCCTCATGCAACCCCTGCCCCCGCTCACCC
ACGGCATGTCTCCCGGCCGCATCTCACCCCCGAGCAGTCTCAGCCCGGACGGCGGCTCGTACTCCCGCAGCGGCAGCCCCATGAGCCCCGGCA
GCCCCGGTTCCCGCGGCTACCGAAGCCTCCCCTACCCACTCCGAAAACGGGACGGCAAAATGCACTACGAGTGCAACGTCTGCAGCAAAACGT
TCGGCCAGCTCTCGAACCTCAAGGTGCACCTGCGAACCCACTCCGGCGAGCGCCCCTTCAAGTGTAACGTCTGCACCAAGTCCTTCACCCAGCT
GGCCCACCTCCAGAAGCATCACCTCGTGCACACCGGCGAGAAGCCCCACCAATGTGATATCTGCAAGAAGCGCTTCTCGTCCACGTCGAACCTC
AAGACGCACCTGCGGCTACACAGCGGCCAGAAGCCGTTCGCGTGCGATCTGTGCCCGCAGAAGTTTACCCAGTTTGTGCACCTGAAGCTGCAC
AAGCGGCTGCACACCAACGACCGGCCGTACGTGTGCCAGGGCTGCGACAAGAAGTACATCAGCGCGAGCGGGCTGCGCACGCACTGGAAGAC
GACCAGCTGCAAGCCAAATAACCTGGAGGAGGAGCTGGCGCTGGCCGCCGCTTCGAACTCCGAGTGTATAGACAAAGAACCGGAGAGCGATCC
ACGAGACTTTTACGAGCTGCAGCACCTCGGGGGCGGCCACCCCCAGTCCCAACCTGGCACCCCGCACCACCACCACCAGCCACCGCACCCCGC
CCAGCTCTGCTCGCCCCAGCTCAGGCACAACCACATCGGATCTCCACCTCCCCACCACCAGCAGCAGCATCCTTCGTGGGTGAGCAACCAGCAC
CACCATCCCGGTGGTCCGGAAATCATCCAAAAAGTGTGCAAAATCGAGCAACATCCCGCGAGCAGTACTACCAGCAGCAGCAACAACAACAAC
AATCCGGCCGTTCCGTCGCACTATTTGACGGGGCCACCCCCGCAGCAGCAGCAAGAGCAGCAGCAGAGTGTTACGAGCCGACCCAGCGTCATC
GAGTCCAGCCAGCCGATGATCATCGAGTGCACGTAG

>*Acromyrmex_echinatior* XM_011062482_1

ATGGAGGCTAGCGAGTGGGATCACGCGACCTTGAGAGAGGAAGAATTCGAGCAGCATGCCGTGTATTTGGTACCGGATGTGTCGGCATCACCGG
GCGATACCAATCGTGCCGAGTCGTCCTTGCCACGGAACCTGGTCCTCAAACCCTCTCAGGCCCTCAACGATGTGTTGGGTGTATGGAGCACCAG
TTACATCCCCAAAGGGACACGTTTTGGCCCTCTGGTTGGTCAGGTGTATACGAAGGATTCGGTACCGGCAGACGCGAACCGAAAGTACTTCTGG
CGGGTATACAAGAACAATGAACTGTTCTATTACATCGACGGTTACGACGTCCAGAAATCGAATTGGATGCGTTACGTGAATCCCGCCTACTCGTCC
GAATCGCAGAACCTAATAGCATGCCAGTATAAGATGAACATTTATTTTTACACGATCAAGCCAATATTACCGCATCAAGAGTTGCTGGTTTGGTAC
TGCAGGGAATTCGCGGAAAGGCTCAATTATCCGCTCACCGGTGAGCTGATGCTTCAGAGAATACGACAACAAGTACAACAATCAACGCTACCAA
CGGAAATTTCGCCCGCCGTTGACGTGATATCACCTCTGAAAGATCCATCAATCTACGAGAGACGTCAGATGACACCAACGGATGGTTCCGTGAG
GTCAGACGAGGGTTACCATTCAAATGGCTATCACGATGAGATTTTGACACCACCAGAAGAAAGCAGTGAGTCCGATAGTGAGAACAACTACGTG
TTGGATTTCAGTAAAAATGCCAAATCATCAGTGTGCAACGATGAAATTCTGAAGGAAGACAACACCGCGGCGAAGAACGAATATAGAAAAGTTA
AGATCAAAATTTCCAAGACCTACGGTAACTTCCAGGCGACAAAGGGCATAGCTGACGGCCCGACGAAGGATAAGGAGCAGGAGTTGTCCAGCC
GAGCGGTCACTCCGGAACTCCAAAAGCCAGTATCACCCGTCATTCTACAGAAAAGCACAGTTCTATCGACTGTGACTGAGGATGAGATATCTGA
GAAGAACATGAAACCTTTCTACGAGCCGGAAGTCAAAGGTGGTAATCTGCCGATGTCCGGCAGATCCGCCTACATAGCTAACCCCAGCAGTTCT
ATTTTGGAGAACATTTTACTACGTAACAGCACAGACAACAACAACAATAGCCACAACCATCATCACAATGGCGGCTCGCAACAACACCATCAGC
ATCATCAGCTGCATCATCAGACTCACGTAGACTCAGTAACGCCGCCACCCTCCAGTCCCACCGAGATGGCATATTCCTACAAGAAGTCTCATCGT
TACGGAAACATTTTGCCCTGCAGCCCGGATTCTAGTTCGAATCTGCCTCTACAAACGGACTCGTGCGTAAACTCGACCAGTGGCAACGGTGGTG
CGCTATCTAGTATGCAGAGTCCCACTGGTAATCTACACTCTAGCACAGGAGGAGGGCTGCATTCGAGCACCGGTGGAAACAACGGCAGCTTACC
GCCGCATCCGGGAAGTTTGCACTCGTCTAGCAATGCCGGTGGCGGCATACATTCCAGCACGAGCGTGCTCTCATCTAGCGCGATGCTAACGTCTA
CCAGCCCGCATTCGGCATCCACAACGGCAAACGGTTGTCTGCCCAAGCGGAAGACTAAATCGCCGTCGCCATCGTCAAGCCAGAACGGTGTATC
CACCCCAACAACGATCCTTTACTCCGGCAGCTCCGGGACCGCTGGCTGTCAGATCGAGTCCGCCAGTCCGGTTTACCCAAACTCCGCTGGCAGT
AGCAGTAGTAGTACCAGCAGTAATCAGAGCATCTCGCCGATTTCGCCGATTCAATCGCCCTCATCCTACAGTCCGTACGGTAGTCTGTACGGTCAT
CAGAATAGTTCGTTGCATCACAATATGGGCTCGTCGTTGTCTATCAACTGCACCGCCTATTCGCCCACGCCGAGCGGCAATGTCGTTTACGAGAG
GACGACGGATGGCAGACGGCTAGAGGCCACTAGCAGTGGCGGCCATCAATTGCCCACCTCATCAACTATGCAAATTGATGGCAATCAGGCATTG
ATCACCGGCACGCACAATCTTCATCTCGGTCATCATCCGGGTCTTACAATTCATCCAAATTCTTCGTCATCTCTCAACAGATACTCACCGGCGAGC
TCCTTATCACCGGACGATCATGGTTGTTCCCAGAGCGGCTCCCTTAGCCCCAATTCCCAGGGTTCGAGGGGTTACAGGTCGCTGCCTTATCCGCT
CAAGAAAAAAGACGGCAAAATGCATTACGAATGCAACGTCTGTTGCAAAACCTTTGGTCAGCTATCAAATCTCAAGGTACACCTGAGGACGCAT
TCTGGCGAAAGGCCCTTCAAATGCAACGTTTGTACCAAAAGCTTCACACAACTCGCGCATCTCCAAAAGCATCATCTCGTGCATACCGGTGAAA
AGCCGCACCAATGCGAGATATGCAAAAAGAGGTTCAGCTCGACTTCGAATCTAAAGACCCACCTTAGACTGCACTCCGGTCAAAAGCCGTACGC
CTGCGACCTTTGCCCCGCCAAGTTCACCCAATTTGTTCATTTGAAACTGCACAAGAGGTTACACACAAACGAACGACCGTACACCTGCCAAGGT

TGCGATAAAAAGTACATTAGCGCGAGCGGCCTCAGGACCCACTGGAAGACAACGAGCTGCAGACCAAATAACATCGAGGACGAGCTCGCGCTC
GCCGCGGCCGTGGGTTCGCCGACATATTACGAGTATGGCCCTGATATGAATGTTGGAAATATGCCCAAGGAATGCGAGTTGGAGCACATTGATAA
TTACGACAGGCATGGATCGACACATGGCCCGCATTCCACGTCCCTCCACAACCTGGAAAATTCCGTGGGTCGACCGAGCGTCATAGAAACGTCC
CAACCACACATTATCGAGTGTACATAAG

>*Megachile_rotundata* XM_012282354_1

ATGGAGGCCAGCGAGTGGGATCACGCGACCTTGAGGGAAGAGGAATTCGAGCAGCATGCCGTGTATTTGGTACCGGATGTGGCGGCGTCTCCG
ACCAATACCGATCGTGCGGAGGCTTCCCTGCCCAGAAATTTGGTCCTCAAACCTTCTCAGGCCCTCAACGATGTACGTTCTTCAGCCAAAGAATT
GTTTCTTGAGACTTCCTTGGTTATGGGAGTATGGAGTACGAGTTACATTCCCAAGGGAACACGATTTGGTCCTCTGGTGGGTCAAGTGTACACCA
AGGACTCGGTACCGGCCGATGCGAACCGAAAATACTTCTGGAGGGTATATAAGAACAACGAGCTGTTCTATTACATCGACGGTTACGACGTACAG
AAATCGAATTGGATGCGTTATGTAAACCCGGCTTACTCGTCCGAATCGCAGAACCTGATAGCATGCCAGTATAAAATGAACATTTATTTTTACACG
ATCAAGACGATATTACCGAACCAAGAGCTGTTGGTATGGTATTGCAGAGAATTCGCGGAGAGGCTCAATTACCCGTTAACCGGCGAACTGATGCT
ACAAAGAATACGACAACAAGTGCAACAATCCGCGTTGCCAAGTGAAATCCCAGCGACGGTAGAAGCAGTATCGCCTCTGAAGGACTCGTCGAT
CTACGAAAGACGGTCACAGATGACCCCGACCGACGGTTCCGTCCGATCGGACGAAGGCTATCACTCGAACGGCTATCACGACGAGATCCTGACG
CCCCCGGAAGAGAGCAGCGAGTCGGACTCGGAGAACAACTACGTGTTGGACTTCAGCAAGAACGCGAAGGCGTCCGTGTGCAGCAACGAGGT
GGTGAAGCAGGACAACGCAGCGGCGAAGAACGAGTACAGAAAGGTGAAGATCAAGATCACGAAGACCTACGGGAACTTCCAGACGTCGAAG
GGTCTGGACGGGAGCGGGAAAGACAAGGATCAGGAGCTGGCGAGCCGAGCGGTGACCCCGGAACTGCCTAAGCCGGTGTCTCCCATCATTCTG
CAGAAGAACTCTGTTCTGTCTACTGTGAACGAGGAAGAAATAGTGGAGAAAAACCCTAAACCGTTCTACGAGTCCGAGGTCAAGGCTAATTTGC
CCAGACCAGCCTATCTGACCAGCCCCAGCAACTCCATCCTCGAGAACATTCTCCTCCGCAGCAGCACCGACAACAACAACAATAACAGCCACA
ATCATCATCACAATGGGACCCAACAGCATCATCAACACCATCAGATCCATCACCAAACGAACGTGGACTCCGTGACACCCCCGCCATCCAGCCC
AACGGAGATGGCCTATTCGTACAAGAAGTCCCATCGTTACGGCAACATCCTGCCCTGCAGCCCCGATTCCAGCTCGAATCTGCCCATGCAGGCGG
ACACCTGCGTCAATTCCACGAGCGGGAACAGTGCCCTGCCGATGCAAAGCCCGACGAGCAATTTGCATTCGAGCACGGGGAACCTCCACTCTA
GTACGGGCAATCTTCATTCCAGCACCGGTCCCAACAACCTTCTGCAATCTCATCCGGGGACCATCCATTCGTCCAGCAACGCGAGCAATCATCAT
TCCAGCGCGAACGTTCTATCGTCCAGCACGATATTAACGTCCACGAGCAACCTCCACTCGTCGACCACGTCCACCAGCAGCTGTCCGCCGAAGA
GGAAGACCAAAAGCCCATCGCCCTCCGCCAATCCCACGGGAGCGTCCACTCCGACGATTCTGTACTCCGGTTCGGCCGCGTGTCAGATAGAATC
GAGCACGGTGTATCCGAACTCCGGTGCGAGCAGCAATCAGAGCGTGTCACCGATTTCGCCGATTCAATCGCCTTCGTCTTACCCGTACGGGATTT
ATCATCAGAACGGATCGTTGCACCACAATGTGGCGCCGTTGTCCATCAACTGCACCGCCTATTCGCCGACTCCTAGCGGAAACGTGGTCTACGAG
AGGACGGACGGAAGAAGGCTGGAGGCTGCCACCAGCAGTCATCAACTACCCACATCGTCCACGATGCAGATCGACACCAATCAGTCGTTGATC
GCTGGCACGCACAACCTCCACCTGGGTCATCATCCGGGTTTGACCATACATGCCAACTCCTCCTCGCTGAATCGATATTCGCCCGCCAGCTCCTT
GTCCCCGGACGATCACGGCTGCTCCCAAAGTGGATCCCTCAGTCCAAACTCCCAAGGATCGAGGGGATACAGGTCGTTACCCTACCCGTTGAAG
AAGAAAGACGGGAAAATGCATTACGAGTGCAACGTATGCTGCAAAACGTTTGGCCAGCTGTCGAACCTCAAGGTACACCTAAGGACTCATTCC
GGTGAGAGACCGTTCAAGTGTAACGTTTGCACCAAGAGCTTCACTCAGCTGGCACACCTGCAGAAGCATCACCTCGTGCACACTGGCGAAAAA
CCACATCAATGCGAGATCTGTAAGAAGAGGTTCAGCTCGACGTCGAACTTGAAAACCCATCTCCGATTGCACTCCGGTCAAAAGCCGTACGCCT
GTGATCTTTGTCCCGCAAAGTTCACACAATTCGTTCATCTGAAGTTGCACAAAAGACTGCACACGAACGAAAGGCCGTACACCTGTCAGGGCTG
CGACAAGAAGTACATCAGCGCGAGTGGCCTTAGAACTCATTGGAAGACGACCAGTTGCAGACCAAATAACATCGAAGATGAGCTGGCCCTTGC
AGCGGCCGTGGGTTCGCCGACGTACTACGAATATGGTGGCCCCGACATGAATGTTGGGAATATGCCGAAAGAGTGCGAGCTGGAGCACATCGAG
AACTACGAAAGGCACGGATCGGCACACGGTCCGCACTCCACGTCCCTCCACAATCTGGAGAACTCTGTGGGACGGCCAAGCGTCATAGAGACC
TCCCAGCCTCACATAATCGAGTGCACCTAA

>*Apis_mellifera* XM_006566009_3

ATGGAGGCTAGCGAGTGGGATCACGCGACCTTGAGAGAAGAGGAATTCGAGCAGCATGCGGTATATTTGGTACCGGATGTGTCGGCATCGCCTG
GCGATACCAATCGTGCGGAGGCATCCCTGCCAAGAAATTTGGTCCTCAAACCTTCTCAGGCCCTCAACGATGTACGTTCCTCCGCCAAAGAATCC
TTTCTCGAGACCTCCTCGGTTTTGGGTGTGTGGAGCACGAGTTACATACCCAAGGGGACGCGGTTCGGTCCCCTCGTGGGCCATGTGTACACCA
AGGACTCGGTGCCGGCCGATGCGAACCGGAAGTACTTCTGGAGGGTGTACAAAAACAACGAGCTGTTCTACTACATCGACGGTTATGACGTCCA
GAAATCAAATTGGATGCGTTACGTAAACCCGGCTTATTCGTCCGAATCGCAGAACCTAATAGCATGCCAGTACAAGATGAACATATATTTTTACAC
GATCAAGCCGATACTACCGAATCAAGAGCTTCTGGTGTGGTATTGCAGGGAGTTCGCGGAAAGGCTCAATTACCCGTTGACGGGTGAACTAATG
CTTCAAAGAATACGACAACAAGTACAACAATCTGCGTTACCGACGGAAATCCCAGCGACCGTGGACGTGGTGTCACCCCTGAAGGACTCGTCG
ATCTACGAGAGACGGTCGCAAATGACCCCGACGGACGGCTCGGTCAGATCGGACGAGGGCTACCATTCGAACGGGTACCACGACGAGATCCTC
ACCCCGCCTGAGGAGAGCAGCGAGTCGGACTCGGAGAACAATTACGTGCTCGATTTCAGCAAGAACGCGAAAACGTCGGTGTGCAGCAGCAA
CGAGGTGGTGAAGCAGGACAACGCGGCCGCGAAGAACGAGTACAGGAAGGTGAAGATCAAGATCACCAAGACGTACGGCAACTTTCAAACGT
CCAAGCCCGGCATGGATGGGAACGGAAAGGACAAGGATCAGGATCAATTGGCCAGCAGGGCGGTGACCCCGGAACTGCAGAAACCCGTTTCG
CCCATTATTCTTCAGAAGAGCGTGGTCCTGACCACGGTCGGGGAGGACGAGATCCCCGAGAAAAACCCGAAACCGTTCTACGAGTCCGAGGTG
AAGGGTGGCGCGTTACCGGTGGCCGCCGCCAGGCCGGCCTACCTCGCCAGCCCGAGCAGCTCCATCCTCGAGAACATCCTTCTTCGAACCGCCA
ACACCGACAACAACAACAACAACAACAACACCACCACCGCCACCACCACCATCAACACCAATCACAATCATCACAACCACGGCGGCAACAAC
GGCCAACAACAGCACCACAACCAGCACCAACATCAAACGATTCACCATCAAACGCACGTGGACTCGGTCACGCCGCCCCCGTCCAGCCCCACC
GAGATGGCGTACTCTTACAAAAAGTCTCACCGCTACGGCAACATTTACCTTGCAGCCCCGACTCGAGCTCCAACCTTCCCATGCAACCGGACA

CGTGCGTCAACTCGACGAGCGGGGGCAACGCCGCTAACCTGCCCATGCAGAGCCCGACCGGCAACCTGCACTCGAGCACGGGCAACCTCCACC
CGGGCCAGGCCGGGCCCAACAATCTTCTACAATCCCATCCGGGGGCGGCCAATATCCATTCGTCCGGCAACGCCTCGACCGGCAACAACCACCA
CTCGGGGGCGGGCGTGCTGTCCTCCACCACGATACTCACGTCCACGAGTAACCTTCACCCCTCGACCGCGTCCAACCAGGCTTGCCCGTCCAAG
AGGAAGACCAAGAGCCCGTCGCCGAACCCGGCCGGGGGCGCGTCCACCGCACCGACCACCATCCTCTACTCGAGCTCGGGGGGATGCCAAATA
GAGTCGAGCCCCGTCTATCCTAACTCGGCGGCCAACGGCAATCAGAGCGTATCCCCCATCTCCCCCATACAATCGCCCTCCTCTTACCCGTACGGT
ATTTACCATCAGAACGGCTCGTTGCACCACAACGTGGCCCCGTTGTCCATCAATTGCACCGCCTATTCGCCGACCCCGAGCGGGAACGTGGTGTA
CGAGAGGGCGGACGGGAGGAGGCTGGAGGCCGCGACGAGCAGCCATCAATTGCCGACCTCGTCCACGATGCAGATAGACGGTAATCAGACGTT
GATAGCCGGCACGCACAATCTTCACCTGGGCCATCATCCGGGCCTCACCATCCACGCCAACTCCTCGTCCACCCTCAACCGATACTCGCCCGCGA
GCTCCTTGTCCCCGGACGACCACGCCTGCTCGCAGAGCGGCTCGCTCAGCCCCAACTCGCAAGGGTCGAGGGGTTACAGGTCGCTGCCGTACC
CGTTGAAGAAGAAGGACGGCAAGATGCATTACGAGTGCAACGTGTGCTGCAAGACGTTCGGCCAACTGTCGAATCTCAAGGTACATCTGAGGA
CTCATTCCGGCGAGAGGCCGTTCAAGTGTAACGTTTGCACCAAGAGTTTCACGCAGCTTGCCCACCTGCAGAAGCATCACCTCGTCCATACAGG
CGAGAAACCGCACCAATGCGAGATCTGCAAGAAGAGGTTCAGCTCGACCTCCAACCTCAAGACTCATCTGAGACTGCATTCCGGCCAAAAGCC
CTACGCCTGCGACCTGTGCCCCGCGAAATTCACCCAGTTCGTTCACCTCAAGTTGCACAAGAGGTTGCACACCAACGAAAGGCCTTACACCTGC
CAAGGCTGCGACAAGAAGTACATCAGCGCGAGCGGTCTTAGGACCCATTGGAAGACGACCAGTTGCAGGCCGAACAATATCGAGGATGAGCTC
GCCCTTGCCGCGGCCGTTGGCTCGCCGACGTATTACGAGTACGGTCCGGACATGAACGTCGGAAACATGCCGAAGGAATGCGAGTTGGAGCAC
ATCGACAATTACGAGAGGCACGGATCCGCTCACGGCCCGCACTCCACGTCTCTCCACAACTTGGAGAATTCCGTGGGGCGGCCAAGCGTCATAG
AGACCTCCCAGCCTCACATAATCGAGTGCACCTAA

>*Camponotus_floridanus* XM_011269305_3

ATGGAGGCCAGCGAGTGGGATCATACGACCTTGAGGGAGGAAGAATTCGAGCAACACGCCGTATATTTGGTACCGGATGTGGCGACATCGCCGA
ACGATACCAATCGTGCGGAAGCATCTTTGCCGCGGAATCTGATCCTCAAGCAATCTCAAGCTCCCAATGTGTTGGGCGTATGGAGTACCAGTTAC
ATCCCTAAAGGGACACGTTTCGGCCCATTGGTCGGTGAAGTATACACGAATGAGTCGGTGCCAGCAGACGCGAACCGAAAATATTTCTGGCGGG
TGTATAAGAACAACGAGCTGTTTTACTACATCGATGGTTATGATGTCCAAAAATCGAATTGGATGCGTTACGTGAATCCCGCCTACTCGTCCGAAT
CGCAAAACCTAATAGCATGCCAGTATAAGATGAACATTTATTTTTACACAATTAAGCCGATTTTACCGAATCAAGAATTATTGGTTTGGTACTGCAG
AGAATTCGCGGAAAGACTCAATTATCCGTTCAATGGCGAGCTAATGCTTCAAAGAATACGACAACATGTACAACAGTCAACCCTACCAGCAGAA
ATCTCACCTACTGTTGACGTGATGCCCTTAAAAGACTCGACGATTTACGAAAGACGTCAGATGACACCAACAGATGGTTCTGTGAGGTCAGATG
AAGGTTATCATTCCAATGGCTATCACGATGAAATTTTGACGCCGCCTGAAGAGAGCAGCGAATCCGATAGTGAGAACAATTATGTGCTGGACTTT
AGTAAGTCATCGGTGTGCGGCAATGAAATTTTGAAACAAGACAATACCGCAACGAAGAACGAATACAGGAAAGTCAAAATCAAGATATCCAAG
ACCTATGGCAACTTCCAGGCAACAAAAAACATAACCGACAGCCCGACGAAGGACAAAGACCAGGAACTGTCTAATAGAGCAGTTACCCCAGAA
CTTCAGAAACCGATCGCACCGATTTCGCCCATCATTCTGCAGAAGAGTACGATATTGTCAACTGTAAACGAAGACGAGATAGCTGAAAAGAAGC
CCTTTTACGAACCGGAGGTCAAAAATAGCAACCTGTCTGCTTCTGGTAGATCTGCCTACCTGGTCAACCCACCCAGTAGTTCTATCCTGGAGAAC
ATTTTACTGCGCAACAGTACGGATAACAATAACAATAACAGCCACGGTCACCATCATAATAGCGGTTCGCAACAACATCACCAACATCATCAGAT
GCATCATCAGACTCACGTAGACTCGGTGACACCGCCACCTTCCAGTCCCACCGAAATGGCGTATTCTTACAAAAAGTCTCATCGTTACAGCAACA
TTCTGCCCTGCAGTCCAGATTCCAGCTCGAACTTGCCCCTGCAAACGGATACGTGCGTTAATTCGACCAGCGGTAATGGCAGTGCGTTGCCCAGC
ATGCAGAGCCCCACCGGCAACATGCACTCTAGTACGGGAGGCCCGCATTCGAGCACCGGAGGAAGCAACGGTAGCCTGCCGATGCATCCCGGG
AGCTTGCACTCCTCCAATAACGGTACCATTCATTCCAGTACCAACGTACTTTCATCTAATGCGATCCTGACGTCTACCAGCAACCTGCACTCGGCA
TCCACGACAATAAACGGTTGTCCGCCAAAGCGAAAGACCAAATCGCCATCACCGTCGTCGAATCAAAGCGGCATTTCTACCCCTACGACGATTC
TTTATTCCGGCAACTCGGGCGCTACCGGCTCGGGAAGTTGCCAAATCGAATCTGCCAGTCCGGTCTATCCAAATTCTGCCGGCAATAGTAATAGC
GGCAGCAGTAGTAATCAGAGCATCTCGCCAATCTCGCCGATCCAATCACCCTCGTCCTATGGGCCGTACGGTAATTTGTACGGCCATCAAAACGG
CTCGCTACATCATAACGTGGAAGCGCCGTTGTCCATCAATTGCACCGCCTATTCGCCCACACCGAGCGGCAACGTCGGCGTTTATGAAAGGTCCA
CGGACGCCAGACGGTTGGAGGCGGCCACCAGCAGCAACGGCCACCAATTACCCACCTCGTCGACCATGCAAATTGACAGTAACCAAGCGTTAA
TCGCAGGAACGCATAATCTCCACTTGGGCCATCACCCAAGTCTCACTATTCACCCTAACTCTTCGTCGTTCCTAAATAGATATTCGCCGGCGAGTT
CTTTATCACCCGACGATCACGGTTGTTCCCAGAGCGGCTCACTCAGTCCCAACTCCCAGGGCTCGAGGGGTTACAGATCGCTACCTTATCCGCTC
AAGAAGAAGGATGGCAAGATGCATTATGAATGCAACGTCTGCTGCAAAACCTTCGGTCAGCTGTCGAATCTCAAGGTGCACCTCAGAACACATT
CCGGCGAAAGACCCTTCAAGTGCAATGTCTGTACCAAGAGTTTCACGCAGCTCGCACACCTTCAAAAACATCATCTCGTACATACTGGTGAAAA
ACCGCATCAATGCGAGATATGCAAGAAGAGATTCAGCTCGACCTCGAATCTGAAGACCCACCTTAGACTACACTCTGGCCAAAAGCCGTACGCC
TGCGATCTTTGCCCCGCCAAGTTCACCCAGTTTGTCCATTTAAAATTGCACAAGAGGCTGCACACGAACGAGCGGCCCTATACCTGCCAGGGTTG
CGATAAAAAGTACATTAGCGCGAGCGGTCTCAGGACTCACTGGAAGACAACAAGTTGCAGACCGAACAACATCGAGGATGAGCTTGCACTCGC
CGCGGCCGTAGGTTCACCAACGTATTACGAATATGGTCCCGACATGAATGTTGGAAATGTGCCCAAGGAATGCGAATTGGAGCATATCGATAATTA
CGACAGGCATGGATCAGCGCATGGTCCGCACTCCACGTCTCTCCACAATTTGGAAAATTCTGTGGGTCGACCAAGCGTTATAGAAACGTCCCAG
CCGCATATTATCGAATGTACATAA

> *Homo_sapiens* NM_182907_2

ATGGAAAAGATCTATTCCAGAGGGGAGCTTCACCACTTCATTGACGGCTTTAATGAAGAGAAAAGCAACTGGATGCGCTATGTGAATCCAGCAC
ACTCTCCCCGGGAGCAAAACCTGGCTGCGTGTCAGAACGGGATGAACATCTACTTCTACACCATTAAGCCCATCCCTGCCAACCAGGAACTTCT
TGTGTGGTATTGTCGGGACTTTGCAGAAAGGCTTCACTACCCTTATCCCGGAGAGCTGACAATGATGAATCTCACACAAACACAGAGCAGTCTA

AAGCAACCGAGCACTGAGAAAAATGAACTCTGCCCAAAGAATGTCCCAAAGAGAGAGTACAGCGTGAAAGAAATCCTAAAATTGGACTCCAA
CCCCTCCAAAGGAAAGGACCTCTACCGTTCTAACATTTCACCCCTCACATCAGAAAAGGACCTCGATGACTTTAGAAGACGTGGGAGCCCCGAA
ATGCCCTTCTACCCTCGGGTCGTTTACCCCATCCGGGCCCCTCTGCCAGAAGACTTTTTGAAAGCTTCCCTGGCCTACGGGATCGAGAGACCCAC
GTACATCACTCGCTCCCCCATTCCATCCTCCACCACTCCAAGCCCCTCTGCAAGAAGCAGCCCCGACCAAAGCCTCAAGAGCTCCAGCCCTCAC
AGCAGCCCTGGGAATACGGTGTCCCCTGTGGGCCCCGGCTCTCAAGAGCACCGGGACTCCTACGCTTACTTGAACGCGTCCTACGGCACGGAA
GGTTTGGGCTCCTACCCTGGCTACGCACCCCTGCCCCACCTCCCGCCAGCTTTCATCCCCTCGTACAACGCTCACTACCCCAAGTTCCTCTTGCC
CCCCTACGGCATGAATTGTAATGGCCTGAGCGCTGTGAGCAGCATGAATGGCATCAACAACTTTGGCCTCTTCCCGAGGCTGTGCCCTGTCTACA
GCAATCTCCTCGGTGGGGGCAGCCTGCCCCACCCCATGCTCAACCCCACTTCTCTCCCGAGCTCGCTGCCCTCAGATGGAGCCCGGAGGTTGCT
CCAGCCGGAGCATCCCAGGGAGGTGCTTGTCCCGGCGCCCCACAGTGCCTTCTCCTTTACCGGGGCCGCCGCCAGCATGAAGGACAAGGCCTG
TAGCCCCACAAGCGGGTCTCCCACGGCGGGAACAGCCGCCACGGCAGAACATGTGGTGCAGCCCAAAGCTACCTCAGCAGCGATGGCAGCCCC
CAGCAGCGACGAAGCCATGAATCTCATTAAAAACAAAAGAAACATGACCGGCTACAAGACCCTTCCCTACCCGCTGAAGAAGCAGAACGGCAA
GATCAAGTACGAATGCAACGTTTGCGCCAAGACTTTCGGCCAGCTCTCCAATCTGAAGGTCCACCTGAGAGTGCACAGTGGAGAACGGCCTTTC
AAATGTCAGACTTGCAACAAGGGCTTTACTCAGCTCGCCCACCTGCAGAAACACTACCTGGTACACACGGGAGAAAAGCCACATGAATGCCAG
GTCTGCCACAAGAGATTTAGCAGCACCAGCAATCTCAAGACCCACCTGCGACTCCATTCTGGAGAGAAACCATACCAATGCAAGGTGTGCCCTG
CCAAGTTCACCCAGTTTGTGCACCTGAAACTGCACAAGCGTCTGCACACCCGGGAGCGGCCCCACAAGTGCTCCCAGTGCCACAAGAACTACA
TCCATCTCTGTAGCCTCAAGGTTCACCTGAAAGGGAACTGCGCTGCGGCCCCGGCGCCTGGGCTGCCCTTGGAAGATCTGACCCGAATCAATGA
AGAAATCGAGAAGTTTGACATCAGTGACAATGCTGACCGGCTCGAGGACGTGGAGGATGACATCAGTGTGATCTCTGTAGTGGAGAAGGAAAT
TCTGGCCGTGGTCAGAAAAGAGAAAGAAGAAACTGGCCTGAAAGTGTCTTTGCAAAGAAACATGGGGAATGGACTCCTCTCCTCAGGGTGCA
GCCTTTATGAGTCATCAGATCTACCCCTCATGAAGTTGCCTCCCAGCAACCCACTACCTCTGGTACCTGTAAAGGTCAAACAAGAAACAGTTGAA
CCAATGGATCCTTAA

>*Mus_musculus* U08185_1

ATGAGAGAGGCTTATCTCAGATGTTGGATCTTCTCTTGGAAAAACGTGTGGGTACGACCTTGCCAAAGGCTGCATTTTAAAACCGTGCTTCTTCA
AGGCAGTCTACTTTACACGGCTTTGGACTCTTACTCAACTGTACAAGCTGCCCCCAAGTCTAGCTCCGGCTCCGTGAAGTTTCAAGGACTGGCA
GAGACTGGGATCATGAAAATGGACATGGAGGACGCTGATATGACTTTGTGGACAGAGGCCGAGTTTGAAGAGAAGTGTACATACATAGTGAACG
ACCACCCCTGGGATTCCGGCGCTGACGGGGGTACTTCTGTTCAAGCCGAGGCATCCTTACCAAGGAACCTGCTTTTCAAGTATGCTGCCAACAA
CAGCAAAGAGGTTATTGGCGTGGTAAGTAAGGAGTACATACCGAAGGGAACACGCTTTGGACCCCTCATCGGTGAAGTCTACACTAATGACACA
GTTCCCAAGAATGCCAACAGGAAGTATTTTTGGCGGATCTATTCCAGAGAGGAGTTCCACCACTTCATTGATGGCTTTAATGAGGAGAAAAGCA
ACTGGATGCGCTACGTGAATCCAGCTCACTCTGCCCGGGAGCAAAACCTGGCTGCCTGTCAGAACGGGATGAACATCTACTTCTACACTATTAAG
CCTATCCCTGCCAACCAGGAACTTCTTGTGTGGTATTGTCGGGACTTTGCGGAGAGGCTCCACTACCCTTATCCTGGAGAGCTCACAGTGATAAA
TCTCACACAAACGGAAAGCAACCCAAAGCAATACAGTAGTGAGAAAAATGAACTCTACCCAAAGAGTGTCCCCAAGAGAGAGTACAGCGTGA
AAGAAATTCTAAAACTGGACTCCAATCCCTCCAAAAGGAAGGACATCTACCGTTCCAACATTTCACCCTTCACTTTAGAAAAGGACATGGATGG
CTTTCGGAAAAATGGGAGCCCCGACATGCCCTTCTACCCTCGGGTGGTTTATCCTATCCGGGCACCTCTGCCAGAAGACTTTTTGAAAGCGTCCC
TGGCCTATGGGATGGAGAGACCCACCTACATAACTCACAGTCCCCTTCCGTCTTCCACAACTCCAAGTCCCCCTGCGAGCAGCAGCCCGGAGCA
GAGCCTTAAGAGCTCCAGCCCCCACAGCAGCCCGGGAAACACGGTGTCACCCCTGGCGCCAGGCCTCCCAGAACACCGGGACTCCTACTCCTA
CTTGAATGTTTCCTATGGTTCCGAGGGCCTGGGCTCCTACCCTGGCTATGCACCTGCCCCCCACCTCCCACCAGCTTTCATTCCTTCTTACAATGC
TCACTACCCCAAGTTCCTGTTGCCACCGTACGGCATTAGTTCCAATGGCTTGAGCACCATGAACAACATCAATGGTATCAACAACTTCAGCCTCT
TCCCTAGGTTGTATCCCGTCTACAGTAACCTCCTTAGTGGCAGCAGCCTGCCTCATCCCATGCTCAATCCAGCTTCCCTACCGAGTTCCCTGCCTA
CCGATGGAGCCCGGAGGCTGCTTCCACCGGAGCACCCCAAAGAGGTGCTTATCCCAGCACCCCACAGTGCCTTCTCCCTTACCGGGGCTGCAGC
CAGCATGAAGGACGAGAGTAGTCCCCCCAGCGGATCTCCAACGGCGGGAACTGCAGCCACGTCAGAACACGTGGTACAACCCAAAGCTACCTC
ATCAGTGATGGCGGCCCCCAGCACTGACGGAGCCATGAATCTCATTAAAAACAAACGAAACATGACTGGTTACAAGACTCTTCCTTACCCTCTG
AAGAAACAGAATGGCAAGATCAAGTATGAGTGCAATGTCTGTGCCAAGACGTTCGGTCAGCTCTCCAACCTGAAGGTCCACCTGAGAGTGCAC
AGTGGAGAACGGCCTTTCAAGTGCCAGACCTGCAACAAGGGTTTTACTCAGCTCGCCCACCTGCAGAAACACTACTTGGTACACACAGGAGAG
AAGCCACATGAGTGCCAGGTCTGCCACAAGAGATTTAGCAGCACAAGCAATCTCAAGACCCACCTTCGATTGCATTCTGGAGAAAAACCTTACC
AATGTAAGGTGTGCCCTGCCAAGTTTACGCAATTTGTGCACCTGAAGCTGCACAAGCGACTGCATACCCGGGAGCGGCCTCACAAGTGTGCCCA
GTGTCACAAGAGCTACATCCATCTCTGCAGCCTCAAGGTCCACCTGAAGGGCAACTGCCCTGCGGGCCCAGCTGCTGGGCTGCCTTTGGAGGAT
CTGACCCGAATCAATGAAGAAATTGAGAGGTTCGACATCAGCGACAATGCAGACCGTCTTGAGGACATGGAGGACAGTGTCGATGTGACCTCC
ATGGTGGAGAAGGAGATTCTAGCTGTGGTCAGAAAAGAGAAAGAAGAAACCAGTCTGAAAGTGTCTTTGCAAAGAAACATGGGGAACGGCCT
CCTCTCCTCAGGGTGCAGCCTCTATGAGTCATCGGACCTGTCCCTCATGAAGTTGCCTCACAGCAACCCACTACCTCTGGTGCCTGTAAAGGTCA
AACAAGAAACAGTTGAACCGATGGATCCTTAA

>*Rattus_norvegicus* NM_001107639_2

ATGTTGGATCTTCTCTTGGAAAAGCGTGTGGGTACGACCTTGGCTGCCCCCAAGTGTAACTCCGGTTCCGTGAAGTTTCAAGGATTGGCAGAGA
CGTCTGGGATCATGAAAATGGACATGGAGGACGCGGATATGACTCTGTGGACAGAGGCCGAGTTTGAAGAGAAGTGTACGTACATAGTGAACG
ACCACCCCTGGGATTCCAGCGCCGACGGGGGTACTTCTGTTCAAGCCGAGGCATCCTTACCAAGGAACCTGCTCTTCAAGTATGCCGCCAACAA
CAGCAAAGAGGTTATTGGTGTGGTGAGTAAGGAATACATCCCAAAGGGAACGAGGTTCGGACCCCTGATCGGCGAAGTCTACACCAACGACAC
GGTTCCCAAGAATGCCAACAGGAAATATTTTTGGCGGATCTATTCCAGAGAGGAGTTCCACCACTTCATCGACGGCTTTAACGAGGAGAAAAGC

AACTGGATGCGATACGTGAATCCAGCTCACTCTGCCCGGGAGCAGAACCTGGCCGCCTGCCAGAACGGAATGAACATCTATTTCTACACTATTAA
GCCTATCCCTGCCAACCAGGAACTTCTTGTGTGGTATTGCCGGGACTTTGCGGAGAGGCTCCACTACCCTTATCCTGGAGAGCTCACAGTGATGA
ATCTCACACAAACAGAAAGCAACCCAAAGCAATACAGCAGTGAGAAAAATGAACTCTACCCAAAGAGTGTCCCCAAGAGAGAGTACAGCGTG
AAAGAAATCCTCAAACTGGACTCCAATCCCTCCAAAAGGAAGGACAGCTACCGTTCCAACATTTCACCCTACACTTCAGAAAAAGACATGGATG
GCTTTCGGAAAAATGGGAGCCCGGACATGCCCTTCTACCCTCGGGTTGTGTATCCCATCCGGGCACCTCTGCCAGAAGACTTTTTGAAGGCGTCC
CTGGCCTATGGGATGGAGAGACCCACCTACATCACTCACAGCCCCCTTCCGTCTTCCACAACTCCAAGTCCCCCTGCAAGCAGTAGCCCAGAGC
AGAGCCTCAAGAGCGCCAGCCCCCACGGCAGCCCGGGGAACACGGTGTCACCCGTGGTGCCAGGCCCCCCAGAGCACCGGGACTCCTACTCC
TACTTGAATGTTTCCTATGGTTCTGAGGGCCTGGGCTCCTACCCTGGCTATGCCCCTGCCCCGCATCTCCCGCCGGCTTTCATCCCCTCCTACAATG
CTCACTACCCCAAGTTCCTGTTGCCACCCTACGGCATCAGCTCCAATGGCTTGAGCACTATGAACAATATCAATGGTATCAACAACTTCAGCCTAT
TCCCCAGGTTGTATCCTGTCTACAGTAACCTCCTCAGTGGGGGCAACCTGCCTCACCCCATGCTCAACCCAGCTTCCCTACCGAGTTCCCTGCCT
ACCGATGGAGCCCGGAGGCTGCTCCCACCGGAGCACCCCAGAGAGGTGCTTGTCCCGGCGCCCCACAGTGCCTTCTCCCTTACTGGGGCTGCA
GCCAGCATGAAGGACGAGAGTAGCCCCCCCAGCGGGTCTCCAACGGCGGGAACTGCGGCCACGTCGGAACACGTGGTACAACCCAAAGCTAC
CTCATCGGTGATGGCAGCCCCCAGCACTGACGGAGCCATGAATCTCATTAAGAACAAAAGAAACATGACTGGTTACAAGACCCTTCCTTACCCG
CTGAAGAAGCAGAACGGCAAGATCAAGTATGAGTGCAATGTCTGTGCCAAGACGTTTGGTCAGCTCTCAAACCTGAAGGTCCACCTGAGAGTG
CACAGTGGAGAACGGCCTTTCAAATGCCAGACCTGCAACAAGGGCTTTACTCAGCTCGCCCACCTGCAGAAACACTACCTGGTACACACGGGA
GAAAAGCCACATGAATGCCAGGTCTGCCACAAGAGATTTAGTAGCACAAGCAATCTCAAGACACACCTTCGACTGCATTCTGGAGAAAAACCTT
ACCAGTGTAAGGTGTGCCCTGCCAAGTTTACACAGTTCGTGCACCTGAAGCTGCACAAGCGACTGCACACCCGGGATCGGCCTCACAAGTGTG
CCCAGTGCCATAAGAGCTACATCCATCTCTGTAGCCTCAAGGTCCACCTGAAGGGCAACTGCCCTGTGGTCCCAGCCCCCGGGCTGCCCTTGGA
GGACCTGACCCGGATCAATGAAGAAATCGAGAGGTTCGACCTTAGCGACAACGCTGACCGTCTTGAGGACATGGAGGACAGCGTCGATGTGAC
CTCCATGGTGGAGAAGGAGATTCTAGCCATAGTCAGGAAAGAGAAAGAAGAAACTGGTCTGAAAATGTCTTTGCAAAAAACCATGGGGAGCAG
ACTCCCCTCCTCAGGGTGCAGCCTCTACGAGTCATCAGACCTGTCCCTCATGAAGTTGCCTCACAGCAACTCACTACCTCTGGTGCCTGTAAAGG
TCAAACAAGAAACAGTTGAACCGATGGATCCTTAA

>*Xenopus_laevis* AF182280_1

ATGAAAATGGATATGGAAGGAATTGATATGACTTTATGGTCAGAGCCTGAATTTGAAGAAAAGTGCACATATATTGTGAAGGATCACTCTTCGGAC
AGCAGCTCAGATGGAAGTAATGTAGTTCAGGCACAAGCATCTTTGCCCAGGAATTTGCTTTTCAAATATGCCTCAGGATGCAAAGAGGTGGTTGG
AGTAGTCAGCAAAGAGTATATACCAAAAGGAACACGGTTTGGACCATTTGTAGGGGAGATCTACACAAATGACACTGTTCCAAAGAATGCCAAC
AGAAAATATTTTTGGCGGATCTATTCAAATGGTGACTTTCAGCACTTCATCGACGGTTACAATGAAGATAAGAGCAACTGGATGCGCTATGTCAAC
CCAGCACACTCTCTTCAGGAGCAAAACCTTGCCGCTTGCCAGAATGGCATGAACATCTATTTTTACACCATTAAGCCTATACCAGCCAATCAGGA
ACTTCTGGTGTGGTATTGCCGGGACTTTGCTGACAGACTTCACTATCCTACTTCTGGAGAGCTTGTAGTGAATCTCCAACAAAGCCTGATCATTAC
AGAGGAGAAAAAGAAAGAACTGATCCCACAGAAAAAAACTCCAAAAAAGGAGCACAGTGTGAAAGAAATCCTAAGGGATACAACAAGCCAC
ATGAAACACAAAGACAATCTACTGAGCTCTATGTCTGCAATCTCACCAGAAAAAGAAAAAGTGGGTGTACATAAAAACTGTAGCCCTGAAAGG
ACTTTCTTCCCAAGGGTAGTTTACCCTTTCCACTCTCACATTCATGAAGACTATTTAAAGGCATCTGTAGGCTATAACACGGACAGACAGAATTAT
CTGATGCATTCACCAATTCAGTCATCCACAACACCAAGTCCTTCATCAAGAAGTAGCCCTGACCAAAGTTTCAAAAGCTCAAGTCCTCACAGTA
GTCCTGGATCGGCAGTTTCACCTCATCATCCAGTGCAAGATCACAAAGAATTTTATCCATTCATTAACAGACCATACAATACTGAAGGTTTGGGGT
CATTTCCAACGTATGCACCCCCAGCAAGTCACCTGCCACCATTTGTATCCTCTTATAACTCAGCGTATTCTAAATATTTATTACCACCCTATGGCATT
GGCTGTAATGGACTCAATTCATTGAACAATATTAATAATTTCAGCCCCTTCTCAAGGATGTATCCAGTTTACAGCAGTATGCTTGCAGGTGGAAGC
CTCCCTCACCATTTGCTAACTCACGCTGCCCTTCCTGGTTCTCTACCACATGAGGGAGGACGTCGATTTCTGCAACCTGAGTTGCCACGAGACTT
TCTTATTCCTGCGCCCAATAGTGCCTTCTCCATCACAGGTGCTGCTGCGAGCATGAAAGACAAACAGAGTAGTCCTACAAGTGGATCACCAACTG
CTGGAACAGCTGCATCCTTAGAGCACATAATGCAACCTAACCCTACCTCAGCAGTGATGTCCACATGCAGTGAAGAAGCCATCAATCTCATTAAG
AGCAAAAGGAATTCGACTGGTTATAAGACTCTCCCATACCCGCTGAAAAAGCAAAATGGAAAGATCAAATATGAATGTAATATTTGCTCAAAAAC
ATTTGGACAACTCTCAAACCTTAAGGTGCATCTTCGGGTACACAGTGGGGAGCGACCGTTTAAATGCCAGACTTGCAATAAAGGATTCACTCAG
CTGGCGCATTTACAGAAGCACTTTTTGGTACACACAGGGGAGAAGCCTCATGAATGTCAGGTTTGTCATAAACGGTTCAGCAGCACAAGCAATC
TTAAGACCCACTTACGACTGCACTCTGGAGAGAAGCCCTACCAGTGCAAACTTTGTCCTGCAAAGTTTACTCAATTTGTTCATCTCAAACTGCAC
AAACGCCTTCACACAAGAGAGAGACCACACAAATGCATTCATTGCCACAAGAGCTACATCCACCTGTGCAGCCTCAACTTCCACATGAAAGGC
AATTGCCCTGTATCTCCAAGATTGGGTTTGTCTAGGGAGGATCTGAATCGAATCAATGACGAAATTGAAAAGTTTGACATCAGTGACAGTGCAGA
CCAGTTAGATGATATGGAAGATATGGACATGTCTCCTGCTGTGGAGAAAGAAATAATGACTCTTCTCAGGAGAGAGATAGATGAATCAAGCATGA
AAGTGTCTGTCTCAAGGAATATGGGCAATGGACTTATCACATCCGGATGCAACTTTTATGACCGATCTGATGCAATAGTAATGAAGTTGCCTCATA
GCTCTCCACTACCTCTGTTACCTGTAAAGGTCAAACAAGAATCCTTTGATCAAATGGACTCTTAA

>*Caenorhabditis_elegans* NM_001264442_1

ATGGGTCAAGGAAGTGGGGATGACGGTGTTCCGCCGGCACCGTTCTCTTCGGCTGCTGCGGCAGCTCACTCACCACCTCATTCTCCCCTTTCTGT
CGGCGTTTCTTCTGCTTCATCTGCCACGTCATCATCTTCCACACCGCCATCATCCACGTCACCTGCCGGAGTTTCAGCGTCAGGTGCGAGAAACG
TTGAAACTGATTGGAAGCAGTCGGGAGATGAGAATCTTGCCGAATTATGTATATTTCATGTTCCAGACAAATCTGTTAGTTTGCCAAATCCAAAGC
GAGCAGAATGCACACTTCCTATGAACCTAATTCTCAAGTCCTCCTCAAAAAAATCGAAAGAAATCGTCAATATGGTCATCTGATCATATTCCGAGAG
GAGTCCGATTTGGCCCGCTCGTTGGAGAAATTCGTTTGGTAGATGTTGACACAGCACTTGTATGTCCTGCTGAAGCATCAATGGCTGGAGGTGGT

CCAGCTCAAGAAGATGTTCCATTTGATGAAGCTCCGGAAGAGTGGAAGATCTATTCTCCATCAGGTGGCAGATTGAATAAAACGATTTGTGTGAA
AGATGATGCGAGATCAAATTGGATGAAATACGTGGCAGCTGCTGAAGAGGAAGATTTTCAGAATCTTGTTGCTGCACAAATTGGAAATGATATCT
ACTTTTACACAGTTAAAAAGATTGAAGCAAATACTGAATTATCATTTTGGTTCAGCAGGGACTACGCAAGGAAATTAAATTATTCAACAAGACCA
TACGTAAGAGTTCGAAGGCCAGCTACTCAACTGATTCCATCTGCACCACCAGCTTCTGCATCAACTGCAATTGCGAGTTTAGCAGAAACAATTGT
GGCAATTGATTATTCAGTTAAAAAACTCATTGAATCACCCATTGATACACTCTCTACTGATGCTTCTTCTGCAAGTGACGAAGAAATGATTGATGT
TGAAGAGCAGGAGAGCTGTACACGGCCTGTTGCAGAAGTCACACGGCCTAATGTTATCCAGAATCCAGTTGTGAGGCCTGTTGCAACCAAAGTC
AATAATTTCCCGGGAATACCCGTTCGACTTGGCAATTTTTATGCAAGTCCGTTGGTGGATTTTAAAGAGTTCATGAGGAAATCCCTTCAATTAAAA
CTAGTTGATACCTCAATGTTTGTGTCCCCTGTTGCTCAAACAACAGCCGCAATAACTGCCACTGGTGGAAGATCCGGCCAGCCGATTGATGTGCA
GCCAGTACTTGCAGCAACTGCAGGAGCTCATTTTGGAAACTATGCAGCAATTTATGGGAGTCAAGATTTCCAGCATGAGCTATCAAAACCTCTGT
ACACATCTGCGTCTCCAGCATTCGGAGGTGGTGGTGGTATGGGTGGAGGATTTGGAATGGGTGGAAGTGCCCATACTTCTTCATTTCATCAACTT
CCATTTGTTAATCATTCGTCTTCATCACACAATGATTCATCATTTAATGGAGTTCCAAATTACGTGCAACAACAGGAAAATGGAAAAACGAGATAC
GCTTGCAAAGACTGCAACAAAACATTCGGACAGCTTTCGAATCTTAAAGTTCATGTCAGAACGCACACCGGCGAACGTCCATTCAAATGTGAAA
TTTGCACAAAAGAGTTCACACAGCTTGCTCATCTTCAGAAGCATCATCTTGTTCATACCGGAGAACGTCCTCATCGCTGTGATATTTGTGATAAGC
GATTCTCGTCGACTTCTAATTTAAAAACTCATCTTCGACTTCACAATGGTCAGAAGCCGTATACGTGTGATGTTTGTGATGCCAAGTTCACTCAAT
ACGTTCATCTAAGACTTCATAAACGTCTGCATGCAAACGAAAGACCGTATAGTTGTGGAACATGTGGAAAGAAATATATTTCACCGAGTGGATTG
AGAACTCATTGGAAAACAACTACATGTAAAGAAGAGGATATGAAAGACTCGATGAGGGATGATTTAATGGATATCAAAGGCGAAATCGATGAAG
GATCAATGTCAGGGTCGGGTTATGGAAATCTGGGAATCTTTGAAAATACACTGAAATACAATCTTCCCAACGCCTCCCTCCTCGGTCAAGGTCCA
AGTGGAATGCAAGAACAACAGGCTCCACCACCAACATCTCAACAGCAGCAACACATGATGTATGGAAACACAATGGGACATATGGGACAAGGA
TCCCATCTTCAGGGGCCACCACCACCTCCACAACATTTCCAAATGGATCATAGTGGAATGCAGAATGGAGGAGGTATTCCACATCAACATCAACT
TATTCAAGGAGGTCCATCATCTGGATCTGGTCAACAGCAACATCCACAACATAATGGGATTCATAGGCTTCCTGATTTGAAAAATCCCCTACTTCC
AAGCCTCGGATTGCCGCATTATCCATAA

>*Danio_rerio* NM_199515_2

ATGTGTGGCTGGGACCAGAGCGTCCTGGCTCCTTATCAAAACTCGGCTATGTTGACTACTGAGGCAGCACCCTACACCGCTGACAATGAGGAGC
TCAAGATGGATGTAGAGGATGTAGACATGACAAAATGGACGGAGGACGAGTTCGAGGAGAAATGCACATACATTGTGAAAGACCACACCTGGG
AAGGACCACTGGAGAACACGGACCTCACCCGGGCTGAGGCGTCTTTACCTAGAAACCTGGCTTTTAAGCACCCGGCAGACTCCAAAGAGGTTA
TTGGTGTAGTAAGCAGAGAATACATCCCTAAAGGCACGCGCTTTGGCCCCCTTGTGGGAGAAAGTTACACTGCAGAAAATGTCCCCAAAGATGC
GAACAGGAAGTACTTCTGGCGGATATACTCAGATGGAGAATTCCATCATTTTGTGGACGGATTGGACGAGGAAAAGAGTAACTGGATGCGCTATG
TGAATCCAGCGCATTCCCAGCAGGAGCAGAACCTTGCCGCCTGTCAAAACGGCATGAACATCTATTTCTACACGGTGAAGGCCATTCCTGCAGA
CCAGGAGCTACTGGTGTGGTACTGCCCTGAGTTTGCCCGCCGTCTCAACTACCCTGCCTCTGGAGAGATTATGATGCAAAAACTCAAGCAGAGC
CTGATTGAAGCCAAGCAGCAAGCAACCGAAGTCAAGCACCCAGTGAAAAGAGAACACAGTGTTTCGGAGATTTTGAAAGATGTGCTGCAAGA
ACCCAGCAGACCACTTCCCACCCGTCCCCGCTGTCCCAAAAGCCCAGACCGACCTCTATACCCCAGTGCTGTCTATCCACCCCGCCCTTCCCTAA
ACGAGGACTTTTTAAAGAGCACCACAGTATTCGGCCTCCCCACACGCTCTCACACACAGTGTTCGGTGACACCCAGCCCGTCAGCACACAGCA
GCCCTGAGAGAAGTCCAGGAAGCAGTCCGACATCAGCATCAATTGAGCCAAGAGAAGCATTTCTCCCTTTCTCTCCAGCACTCTACAATCGTGG
CCTGAACCACTACCCAGGATACTCACCAGCAGGCTCCATGCCATTTACCCAAACCCGCACTACTCGCGCTACCTCATGCACCACTACCCTGTTA
GTAGTTTGAGTGGGCCATCTACATTGGGGGGTATATTTCCACACATGTATCCATTTTACAGCAGCTTGGTACCTCCTCATGTACCATTCCCACCTTC
CATGCTTTCATCCGAGGGCAGTCGTCAGTTTATGCTTCCACCTGACTCTCCGGCCCCCAGGGACATTCTGCTTCCAGCAGCCACCAGTGCCTTTT
CTGCAGCCACTTCTCTGAAAGACAAACCCCTGCATGGGCATGGACACCCTTACGCTCCTGCTGGTGGATCTCCAACAGCCGGCTCTGCGGCCTC
TACGGGATGCATTCCCACCAAGCCTACCTCTGCAATTCTGAGCACCAGCCGCTCAGAGGATGAGGCCATCAATCTGAGCAAGATGAAACGAGGT
TCCACAGGCTACAAAAGTCTTGATTACCCACTTAAGAAGCAGAATGGGAAGATCAAGTATGAGTGCAATGTTTGCACCAAGACTTTTGGTCAAC
TTTCTAATCTTAAGGTGCACCTGCGTGTCCATAGTGGGGAACGACCCTTTAAGTGTCAGACCTGCAATAAAGGTTTCACTCAGCTCGCCCACCTG
CAGAAACACTATTTGGTCCACACAGGAGAGAAACCCCACGAGTGCCAGGTGTGTCACAAGCGTTTCAGCAGCACTAGCAACCTGAAAACCCAT
TTGCGATTACATTCTGGGGAAAAGCCCTACCAGTGCAAGATCTGCCCAGCCAAGTTCACACAGTTTGTCCACCTCAAGCTGCACAAGCGGCTGC
ACACGCGAGAACGTCCACACCAATGCCCACATTGCCACCACAACTACATTCACCTCAGCAGCCTACGGTTCCACCTCAAGGGCTACTGCCTCGC
AGTCTCTCCTTCTCCAAGCTGCAGCTTGGACGAACTCAACCGAGTCAACGAGGAAATTGAACGCTTTGACATCAGCGATAATGCCGACCGGCTA
GAAGAAATGGAAGGGTTTGATGTGGAGGGCATGGTGGAGAAGCAGATATTTGGCCTGCTCTGGCAGGAGATGGATTTTAAGGCGTCTTATCACA
AAGGTAGCACTGGAGGGGACCTGCACCCCCGAGCGCCTGCATTATCCGCGTATCATCTGAATGAGCATGACAGTGAAGCTTCTGTCATCAAAGTT
CATCGCAGCAGCCCGATCCAACTCCTACCCATCAAGGTGAAAAAGGAGACAGAGGAGGCCATGGATACCTAG

>*Papilio_machaon* XM_014508175_1

ATGAGACTGTCAACAAGCGAAGAAGTTCCGAGCACAACTGTGGTCGGCCGCACCGGTAGCCCGGGCGGCGGAAGTGCTGCATCGGTCGCAGC
AGTGGTCGGAACTGGCGCTTACGATGTGACGAGGATGAGGGAGGAGGAGTTTGAGAGGCTCACTGTATACATAGTGCCTGACGTGCCGTGCGA
GAGAGGGACCCCCAACAGAGCCGAGAAGACCTTACCCAGAAGCCTCGCACTAAGACCCTCCGTAGTGCTATCAACTCCTAATACACCGACCGA
AGGAGTATGGAGTACGGGCGTTATTCCCCGCGGCACACGCTTCGGCCCGTTCGAAGGCACCCGAACGCCAAACAAACCTAATGATAAAGTTTCC
TGGCGATACTATTGGAGGGTACAGGAAACTGTCAGACATCATACTTATGGAAATATTAAGATATTTAAGGATAGTGATTACTATTACCTGGACGGAT
CGGACACGAGTGTTGCAAACTGGATGCGTTACGTGGCCTCTGCCTACTCCCTCTCCGTGATGAACCTCGTGGCTTGCCAGCATCAAGAGCACATA

TACTTCTACACAATCAGAGACATTATGCCAAATGAAGAATTAATGGTATGGTACTGCAAAGACTTTGCGACGAGGCTAGGATACGATATCGACCCT
GAAAGAGCAACCTATTCCATCTGCAAAGAGGAAACTATCAAAAAAGCATACGGCATACCCCCTGCTCCAGTTCATCCCGAGATTGCATTTAACCA
CATGAAGTACGTGCTCGGCTTAACAAACCCTAAAGAACTCCTGGAAGCTGATATTCCTAGCCGAAGAATAAAACGTGAATCTATTGAAAAGGCA
CACATTGAAAATTACACACCACGTGAAGATCCTCGAATAACCAATAAGAATGAAAGGACAATTATAAGAACTGATGTATCGGGTAATATTATGCAT
CACAAATCAACAAATGATATTCAAATGGCCAGTGAACATAGAACGTTATCACCCAATGGTCTTATGCAGCCCCCATCACCAGTCAAAATGCACCA
CAGAGACAATCAAACTTTAATAATTCATCAACACAAAGAGAGTACATCACATGTAGTTATACATCAACTCGATAATCCGAGATTACATGCTAACAG
GTCACCAGGTCAAAACATACATATGAGAGATATGCGAGGACCTTCACCGATGTTACAACATCACCAGGACAAATTAGGGAGTAAATCGCCAGGT
CTAACGCACTGCAGTGCTCAACACTATGAACATCAACTGACGCCCAATGACGGATCGGTAAGGTCAGATGAAGGTTACCACAGCAATGGATATC
ATGACGAATTTACACCACCCGAGGATTCCAGCGATTCAGACGTAGACTCCAACTATGTTCTTGATTGCTCGAATAAAACAAATGAACCAAAAGAG
ACTGTTATAGTGCAAAAAATGGATCAAGATATGCATAGAAATGAATACAGAAAAGTTAAAATTAAAATGCCTAGGGCATACCAATACCAGCATCA
CAGGGACATGGACTGCGACAGTGAAAAAGAACTGATTATTGCGGAAGACGTAGCAAACTTATCGCCACAGAACTTATCTCCGCCCAGGCGCGA
CCCGGCTACTTCCACTGTAATAGTCCTAGAATCGTCACAAAATACTGTTGTTCCTATTAACAAGCCTTATTATGAAGATGGTACGATATCTCCAACA
CCGCAACCAGCATTCATGAGATACTCACCGCCTGACACAAGGATATTGGAAACCATATTAACTGGAAGCAGAATCGATACAAATAACAACGATCC
TAATCGCCGACAACCTAACGCGACGCCACCACCATCGTCCCCTACTGAAATGGCATATTCATACAAAAAAAGTCAGAGGTACGGGAACGCCTGC
AGTCCTGATTCTAGTTCTAATTTAACACCCTTGCCATCACTGCTGCCTTTACCACAACAATTACCGGCTCCCAACGCTACGTCAGTCTATTCGTCGT
CCCCGCCGCACTCTATCCCACATACGACAGAAATTCGGGAAATAAGAGAAATACGCGAGATAAGAGAGACTAGATACGAAAGTCACTACCCAAA
TTACTCGTACAATTTATACTCGCCACCAAACTCTACTATTATCACGCAACTTGGATCACCGCCTAGCACCTATTCGCCAACCGTGTCTTCCACTCAT
CAGTATGAAAGGTCACAAAGCGCTCAAAGCAATCATCACTATCCCTCCTCGACCAGCGTTATAACACTTAAAAACTGTTCACAATTGAGTTTAAT
ACAAAACTCAAATGTCAATGGTCAACTTGTACCACAACACGGCAGCATGAGTCCGGATGGCACTTGCAGTCTCATGGTCGCCCCGATGAGTCCA
AACTCACAGTCCTCTCGCGGTTACAGAAGTCTGCCGTATCCATTAAAGAAGAAAGATGGAAAAATGCACTACGAATGTAACGTTTGCTGTAAAA
CGTTCGGCCAACTCTCGAATTTAAAAGTACATTTGCGGACACACTCCGGGGAACGGCCGTTCAAATGTAACGTGTGCAATAAGTCCTTCACGCA
GCTTGCACACTTACAGAAACACCATCTCGTACACACCGGAGAAAAACCACACCAGTGCGACATCTGCAAGAAGAGATTTTCATCCACTTCAAAT
TTGAAGACACATTTAAGACTACACTCAGGTCAAAAACCTTATGCTTGTGATTTGTGTATGCAAAAGTTCACTCAATTCGTCCATTTGAAACTTCAC
AAGCGGCTGCACACGAACGACAGACCATACGTCTGCCAGGGGTGCGACAAAAAATATATAAGCGCATCCGGACTAAGAACACATTGGAAAACC
ACAAGCTGTAAACCCAATAACATCGAAGAAGTTTTAGCAATCACCAACGCTGCGAACACTGAATGCATGGGTTCCATCGACAAAGACTGCCACG
ATAGAGAGGGGCACGAGGCGTACGAGGTGCATTCCGCTTCACAGGCGGGCCTCGCGGGAGTGGGTGGCTCGCGGCTGCTGGCAGGCGAAGGC
GCGCTTGGTGGTCACGCGCATTCCACCACACCGCACCTCGCACCGGGCCCGCTGTCCCCGCCGCACACTCAGCACACTCAGCACACTCAGCAC
GCGCAGCACACGCAGCCCTACCGCAAGATGCACGGGCAGGGGCAGGAGCACGAGCCGGCGCCGCAGCACTACTCCTCGGCGGGAGAGACGCG
CCCCAGCGTGATAGAGTCGAGCCAGCCACTCATCATTGAGTGCACCTGA

>*Papilio_polytes* XM_013285979_1

ATGAGACTGTCAACAAGCGAAGATGTTCCGAGCACGACTGTGGTCGGCCGCACGGGTAGCCCCGGCAGCACAAATGCTGCATCGGCGGCAGCA
GTGGTCGGAACCGGCGCTTACGATGTGACGAGGATGAGGGAGGAGGAGTTTGAGCGGCTCACTGTATACATAGTGCCTGACGTGCCGTGTGAG
AGAGGGACGCCCAACAGAGCAGAGAAAACCTTACCCAGAAGCCTCGCATTAAGACCCTCCGTAGTGCTATCAACGCCTAATACACCGACTGAA
GGAGTATGGAGTACGGGCGTTATTCCCCGCGGCACTCGCTTCGGCCCGTTTGAAGGCACAAGAACGCCTAACAAACCCAACGACAAAGTTTCCT
GGCGGTACTATTGGAGGGTACAGGAAACTGTCAGACATCATACTTATGGAAATATTAAGATATTTAAAGATAGTGATTACTATTACCTGGACGGATC
GGACACGAGTGTTGCGAACTGGATGCGTTACGTGGCCTCTGCCTACTCCCTCTCTGTTATGAACCTCGTGGCTTGCCAGCATCAAGAGCACATAT
ACTTCTATACAATCAGAGATATTATGCCAAATGAAGAATTAATGGTTTGGTACTGCAAAGACTTTGCGACAAGGCTAGGATATGATATCGATCCTG
AAAGAGCAACTTACTCAATTTGTAAAGAGGAAACTATCAAAAAAGCATATGGCATACCCCCGGCTCCAGTTCATCCGGAGATTGCATTTAACCAC
ATGAAGTACGTGCTCGGCTTAACGAACCCTAAAGAACTTCTAGAAGCTGATATTCCCACCCGAAGAATAAAACGTGAATCTATTGAAAAGGCAC
ACATCGACAATTATACACCACGTGAAGATCCTCGAATAACAAATAAGAACGAAAGGACAATTATAAGAACCGATGTATCGGGTAACATTATGCATC
ACAAATCAATAAGTGATAACCAAATGGCCAGTGAACATAGAACGTTATCGCCGAATGGTCTTATGCAGCCTCCGTCACCAGTCAAAATGCACCAT
AGAGACAATCAGACTTTAATCATTCATCAACACAAAGAAAGTACATCGCATGTAGTAATACATCAACTTGATAATCCTAGATTACATGGCAACAGG
TCACCAGGTCAAAACATACATATGAGAGATATGCGAGGACCTTCTCCGATGTTACAACATCACCAGGACAAACTAGGAAGTAAGTCACCCGGCT
TAACTCACTGCAGCGCTCAACACTATGAACATCAACTGACGCCAAATGACGGTTCAGTAAGATCAGATGAAGGCTACCACAGCAATGGTTATCAT
GACGAATTTACACCACCCGAGGATTCCAGCGATTCAGACGTAGATTCCAACTATGTTCTTGATTGCTCGAATAAAACAAATGAACCAAAAGAAAC
TGTCATAGTGCAAAAAATGGATCAAGATATGCACCGAAATGAATACAGAAAAGTTAAAATAAAAATGCCAAGAGCATACCAATACCAGCATCACA
AAGACATGGACTGCGACAGTGAGAAAGAACTGATTATTGCGGAAGACGTGGCTAACATCTCGCCACAAAACTTATCTCCGCCGAGGCGTGACCC
AGCCACTTCTACTGTAATAGTCTTAGAATCGTCACAAAACACAGTTGTTCCTATCAATAAGCCTTATTATGAAGACGGTACAATATCTCCAACACC
ACAACCGGCCTTCATGAGATACTCTCCGCCTGATACACGGATATTAGAAACCATTTTAACTGGAAGTAGAATGGATACAAATAACAATGATCCCAA
TCGCCGTCAACCTAACGCGACGCCACCACCATCGTCTCCTACTGAAATGGCATATTCATACAAAAAGAGTCAAAGGTACGGAAACGCCTGCAGC
CCAGATTCTAGTTCCAATTTAACACCCTTGCCATCACTGCTGCCTTTACCACAACAATTACCGGCGCCCAACGCTACCTCCGTCTACTCATCATCG
CCGCCGCACGCTATCCCACATACGACAGAAATTAGAGAAATACGAGAAATACGCGAAATAAGAGAGACAAGATATGAAAGCCACTACCCAAATT
ACTCATACAGCTTATACTCACCACCCAATTCTACTATTATCACGCAACTTGGATCACCGCCTAGCACTTATTCACCGACCGTGTCATCCACTCACCA
ATATGAAAGGTCACAAAGCGCTCAAAGCAACCATCACTATCCTTCGTCGACTAGCGTTATCACACTTAAAAACTGCTCACAGTTGAGTTTAATAC
AAAACTCAAACGTCAATGGTCAACTGGTACCACAACACGGTAGCATGAGTCCAGATGGCACTTGCAGCCTTATGGTCGCCCCGATGAGTCCAAA
CTCACAGTCCTCTCGCGGATACAGAAGTCTTCCTTATCCATTAAAGAAAAAAGACGGAAAAATGCATTACGAATGTAACGTTTGTTGTAAAACAT

TCGGTCAACTCTCGAATTTGAAAGTGCATTTGAGGACACACTCCGGAGAACGGCCTTTCAAATGTAACGTGTGCAATAAGTCCTTCACGCAGCT
TGCGCACTTACAGAAACACCATCTCGTGCACACTGGAGAAAAACCTCACCAGTGTGACATTTGCAAGAAGAGATTTTCATCCACTTCAAATCTG
AAGACACATTTAAGACTGCACTCAGGCCAAAAACCATACGCCTGTGATCTGTGTATGCAAAAGTTCACTCAATTCGTCCATTTGAAACTCCATAA
GCGACTGCACACGAATGACAGACCTTATGTGTGCCAGGGGTGCGACAAGAAGTATATAAGTGCATCCGGACTAAGAACACATTGGAAAACCACT
AGCTGTAAACCTAATAACATCGAAGAAGTTTTAGCAATCACCAATGCTGCGAACACTGAATGCATGGGTTCTATGGACAAAGACTGCCACGATAG
AGAGGGGCACGAAGCGTATGAAGTGCATTCGGCGTCGCAGGCGGGTCTCTCAGGAGTGGGTGCGCGACTGCTGGCCGGCGAGGGCGTGCTAG
GTGGGCACGCGCACTCCACCACGCCGCACCTCGCGCCGGGCCCGCTGTCGCCGCCGCACACGCAGCACTCGCAGCACTCGCAACACGCCCAGC
ACTCGCAACATGCCCAACCATACCGTAAGATGCATGGGCAGGCGCAAGAGCACGATCCGGCACCGCAGCATTATTCCTCGACGGGCGAGACGCG
CCCCAGCGTCATAGAGTCGAGCCAGCCACTCATCATTGAGTGCACTTGA

Table S2. The microarray data of *BmBlimp-1* in the silkworm.

A. Microarray data of silkworm *BmBlimp-1* gene expression in larval tissues

**Tissue**	Ovary -1	Ovary-2	Ovary -3	Ovary -4	Ovary -5	Ovary -6	Testis -1	Testis -2	Testis -3	Testis -4
**value**	36	162	105	79	182	325	396	590	502	311
**Tissue**	Testis -5	Testis -6	Head-F -1	Head-F -2	Head-M -1	Head-M -2	Integument-F -1	Integument-F -2	Integument-M -1	Integument-M -2
**value**	705	379	432	436	795	654	214	208	268	338
**Tissue**	Fat-F -1	Fat-F -2	Fat-M- 3	Fat-M- 4	Midgut-F -1	Midgut-F -2	Midgut-M -1	Midgut-M -2	Blood-F -1	Blood-F -2
**value**	41	95	90	119	93	299	433	62	72	97
**Tissue**	Blood-M -1	Blood-M -2	Malpighian tubes-F -1	Malpighian tubes-F -2	Malpighian tubes- -3	Malpighian tubes-F -4	Malpighian tubes-M-1	Malpighian tubes-M -2	Malpighian tubes-M -3	Malpighian tubes-M -4
**value**	97	64	160	30	226	115	167	217	272	43
**Tissue**	A/MSG-F -1	A/MSG-F -2	A/MSG-F -3	A/MSG-F -4	A/MSG-F -5	A/MSG-F -6	A/MSG-M -1	A/MSG-M -2	A/MSG-M -3	A/MSG-M -4
**value**	193	98	153	130	125	223	176	168	168	150
**Tissue**	A/MSG-M- 5	A/MSG-M- 6	PSG-F -1	PSG-F -2	PSG-F -3	PSG-F -4	PSG-M -1	PSG-M -2	PSG-M -3	PSG-M -4
**value**	259	142	54	40	62	54	23	57	33	66

B. Microarray Data of *BmBlimp-1* from Larvae to Adult Stage.

	5th4D	5th5D	5th6D	5th7D	W0	W12h	W24h	W36h	W48h	P0	P1	P2	P3	P4	P5	P6	P7	P8	Moth
**Female**	0	0	0	0	0	0	0	0	0	0	0	0	2.46	0.00	0.00	7.97	11.97	7.52	7.69
**Male**	0	0	0	0	0	0	0	0	0	0	0	0	4.62	10.09	0.00	15.68	17.88	8.35	14.10

Table S3. The list of predicted target genes, overlapping genes and GO annotation.
(This data is attached to an Excel file)

Figure S1. The titer of ecdysone and the expression level of the *BmBlimp-1* gene in the pupal stage. The red line indicates the expression level of *BmBlimp-1* in female; the blue line indicates the male, and the dotted line indicates the titer of 20E.

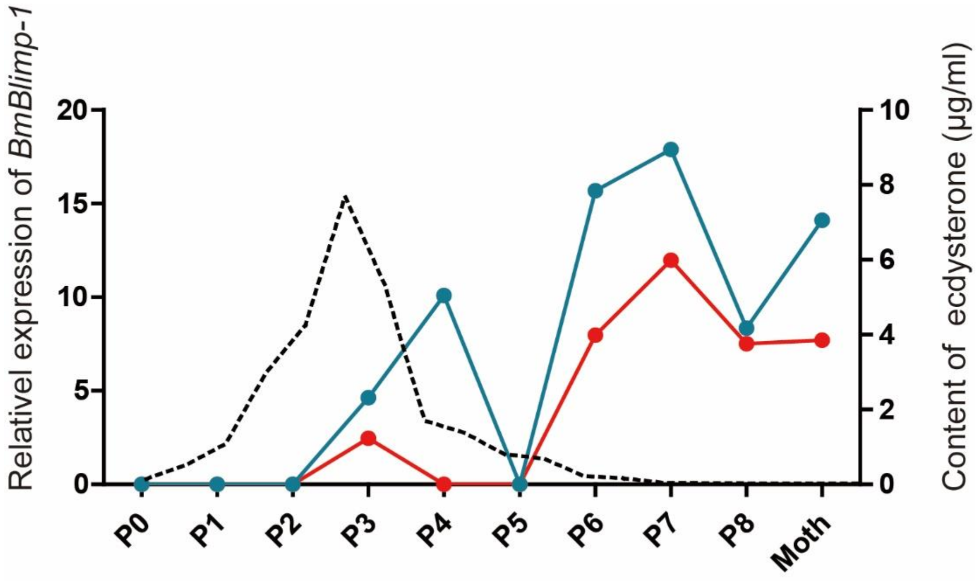

Table S4: The list of primers used in this study.

Name	Forward	Reverse
BmBlimp-1 for cloning	ATGTTGACTCACTGTGGTGGAC	TCAGGTGCATTCTATGATGAGT
BmBlimp-1 for qRT-PCR	GGAACTCAATACCGTCCCTTAG	TGGCTGGATTCTATAACGCTAG
WCP1a	CTCCTGTCGCTAAGGTGATC	AGAGTAGGCGAAGTCGTATTTG
WCP1b	TCTAAAATCGTAGCCTTGACCG	CTACATATTTGGTGGCATGGAG
WCP2	CAACTACTCAGTAGCTGATGGT	ATGAACCCTTCACTACATCACC
WCP3	AGTAGCCAAAATCGTAGCTCAT	CTTCTTGTTGGGACTTGTTGTC
WCP4	ACATATCATACAGCTCCAGCAA	GAGTATCCGTATTTAGGGTGGG
WCP5	CATCAAGCAACGGCACATTATA	CTCGACGGAATATGAGAACTCA
WCP6	CTCATCTCATACACGCTAGTCC	AGTCACCAGTTAGACTGTCTTG
WCP7	TTGTAGTGACAGTCGTGAGTAC	TGCTACTGGTACAACATGGTAG
WCP8	GAGAGTATAGTCTTCACGAGGC	AGTCCGGAGTTGATAACTTCAG
WCP9	TGAAGAGCAGGGCTACATAAAA	TAGAGCTTTTTGTATCTCGGCT
WCP10	CCAGTGGAAACCTCAAAATACG	TTGTTGTGGTCGAAGTCTGATA
WCP11	CCTAAGATCGTTCGGTCTGAAT	GTGTTCGTGTAACTGTAGCTTC
chitin synthase A	CGAGGATTTATGCTTGTGCTAC	TACACGAAGATACTTCTGAGCG
Wnt1	GGACTTGTTTGTTTCCCCTCG	GGTTATTAGCCCTCTCTTGTATTGC
decapentaplegic（*dpp*）	GTACCCTCTGATGAAGTAGCTC	CACGGAATCGAGAAGTCTTAGA
engrailed (*en*)	AAGATCTGGTTCCAGAACAAAC	GTTCCTCCTCTTCCTTTGTCAG
apterous (*ap*)	GGACCATGAAATCTTACTTCGC	AAGTATGACCATTAACCCCGTT
Serrate (*Ser*)	GATGTAACGCATGTTGGTGTAG	CTTCGTCTCCGATCCATTCATC
asense (*ase*)	ACAAAGAAAACACGTCTCGTTC	GCAGGCGAACTAATCATGAAAT
cubitus interruptus (*ci*)	CGAGATAACGTGAGCAATAAGC	GGTTTCGATAAAATCTCCTGGC
Serum Response Factor (*SRF*)	GATTCTGGGAAAAGGCTGATCC	AATTGCCTCACCTTCATATCCT
RPL3	CGGTGTTGTTGGATACATTGAG	GCTCATCCTGCCATTTCTTACT
